# Trimethyl-Substituted Carbamate as a Versatile Self-Immolative Linker for Fluorescence Detection of Enzyme Reactions

**DOI:** 10.3390/molecules25092153

**Published:** 2020-05-05

**Authors:** Noriaki Nakamura, Shohei Uchinomiya, Kazuya Inoue, Akio Ojida

**Affiliations:** Graduate School of Pharmaceutical Sciences, Kyushu University, Fukuoka 812-8582, Japan; jokiuhj@gmail.com (N.N.); uchinomiya@phar.kyushu-u.ac.jp (S.U.); inoue.kazuya.751@s.kyushu-u.ac.jp (K.I.)

**Keywords:** fluorescent probe, self-immolative linker, trimethyl carbamate, enzyme detection

## Abstract

Self-immolative linker is a useful building block of molecular probes, with broad applications in the fields of enzyme activity analysis, stimuli-responsive material science, and drug delivery. This manuscript presents *N*-methyl dimethyl methyl (i.e., trimethyl) carbamate as a new class of self-immolative linker for the fluorescence detection of enzyme reactions. The trimethyl carbamate was shown to spontaneously undergo intramolecular cyclization upon formation of a carboxylate group, to liberate a fluorophore with the second time rapid reaction kinetics. Interestingly, the auto-cleavage reaction of trimethyl carbamate was also induced by the formation of hydroxyl and amino groups. Fluorescent probes with a trimethyl carbamate could be applicable for fluorescence monitoring of the enzyme reactions catalyzed by esterase, ketoreductase, and aminotransferase, and for fluorescence imaging of intracellular esterase activity in living cells, hence demonstrating the utility of this new class of self-immolative linker.

## 1. Introduction

**Figure 1:**
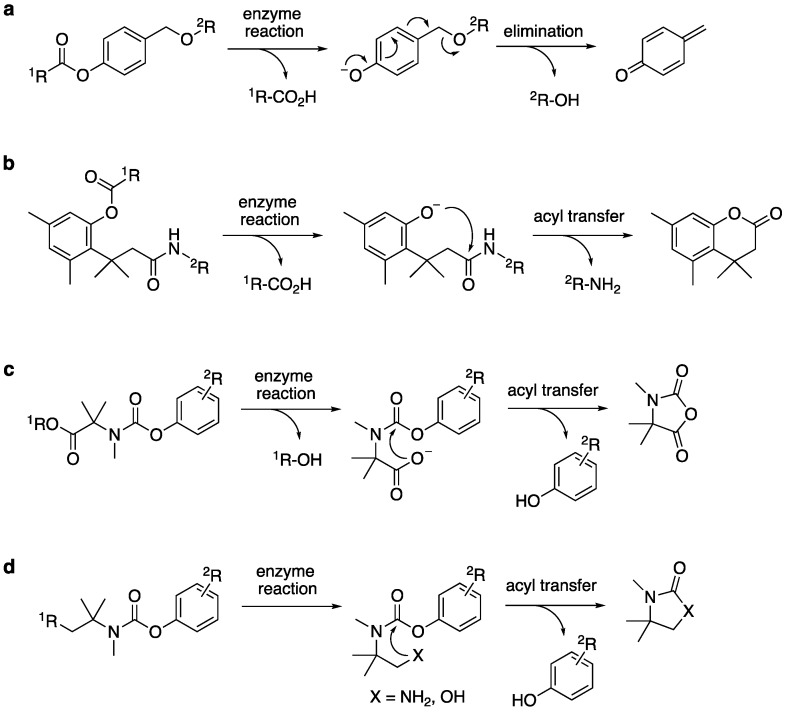
Activation mechanisms of self-immolative linkers: (**a**) 1,6-elimination to form quinone methide, (**b**) acyl transfer of trimethyl lock, and (**c**,**d**) acyl transfer of the trimethyl carbamate developed in this study.

Fluorescent probes are invaluable chemical tools for the detection of enzyme activities in biological research. To date, a variety of small-molecule fluorescent probes have been developed to detect various enzyme reactions, such as esterase/protease-catalyzed hydrolysis and oxidoreductase-catalyzed redox reactions [1,2,3,4]. These probes are, in most cases, designed as fluorogenic substrates that emit bright fluorescence in response to enzyme-mediated structural change. To achieve precise and sensitive detection in biological systems, the probe should serve as an enzyme substrate and be efficiently converted to a fluorescent product. In addition, the probe should be sufficiently stable under biological conditions to avoid non-enzymatic degradation, such as hydrolysis, which would generate unwanted background fluorescence signal. To fulfill these requirements, several types of self-immolative linker have been devised and exploited as a building unit in probe design [5,6,7]. In this molecular design, self-immolative linker is inserted between fluorophore and reactive group as a functional adaptor. Upon enzymatic transformation, structural change on the reactive group triggers spontaneous cleavage of the linker unit to liberate a fluorophore with bright emission. Representative self-immolative linkers, such as *p*-hydroxybenzyl group and trimethyl lock system are shown in Figure 1a,b [8,9,10]. Although these linkers have been widely used in fluorescent probe design, there is still need for development of new type of self-immolative linker, which would allow us to sense various types of enzymatic reactions with enhanced enzyme selectivity, higher reaction kinetics, and greater chemical stability. In this study, we report a new class of self-immolative linker useful for fluorogenic detection of enzyme reactions. Fluorescence-based study revealed *N*-methyl dimethyl methyl (i.e., trimethyl) carbamate as an auto-cleavable linker (Figure 1c), which is rapidly cleaved from the fluorophore upon generation of a free carboxylate. Interestingly, this auto-cleavage reaction was also induced upon formation of an amino and hydroxylate group on the trimethyl carbamate unit (Figure 1d). Utility of this linker was demonstrated by the fluorescence monitoring of various enzymatic reactions and by the fluorescence imaging of intracellular esterase activity in living cells.

## 2. Results

### 2.1. Development of N-Methyl Dimethyl Methyl Carbamate Linker

**Figure 2:**
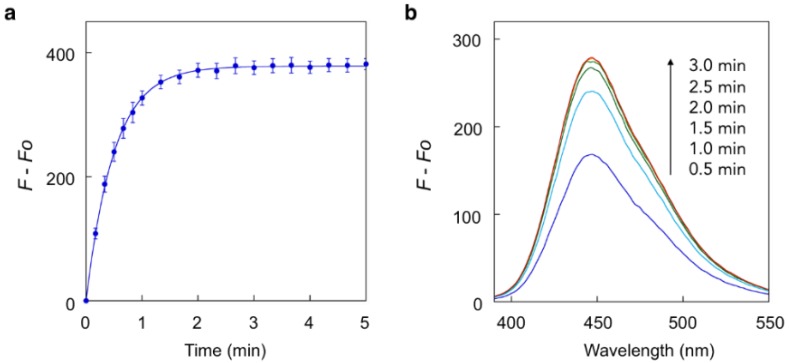
Fluorescence analysis of spontaneous degradation of **5**. (**a**) Time-trace plot of the fluorescence intensity of **1** (λ_em_ = 450 nm) released from **5**. (**b**) Fluorescence spectral change of **5**. Measurements were conducted at 0.5, 1, 1.5, 2, 2.5, and 3 min after addition of **5** into the neutral buffer solution. Measurement conditions: (**5**) = 5 μM, 50 mM HEPES, pH 7.4, 37 °C, λ_ex_ = 320 nm.

Our study first focused on the development of a carboxylate-appended carbamate linker, which would be cleaved by the intramolecular acyl transfer reaction (Figure 1c). To this end, we synthesized a series of coumarin carbamates, bearing different carboxylate units, and evaluated the rate of spontaneous degradation under neutral aqueous conditions (50 mM HEPES buffer, pH 7.4) by fluorescence measurement. The reaction kinetics parameters of coumarin probes are summarized in Table 1. The non-fluorescent carbamate probe **3**, bearing glycine linker, showed slow increment of fluorescence over 24 h, suggesting the spontaneous release of 7-hydroxycoumarin **1** to be sluggish (half-reaction time: *t_1/2_* = 30 h). However, we found that the introduction of methyl group(s) at α-position of the carboxylate group greatly facilitated the release of **1**, and the half-degradation time (*t_1/2_*) of α-methyl carboxylate **4** was reduced to 0.58 h. This effect was more prominently observed in α,α-dimethyl carboxylate **5** (Figure 2), which showed over 5000-fold enhancement of reaction rate (*t_1/2_* = 21 s) than in **3**. The large rate enhancements in **4** and **5** could be reasonably attributed to Thorpe-Ingold effect [11,12,13], which contributed to increase the population of carboxylate group properly oriented for intramolecular cyclization due to reactive rotamer effect. In contrast, the corresponding methyl ester **6** stably existed under neutral conditions, strongly suggesting the participation of carboxylate group of **5** in the auto-cleavage reaction. Compound **7,** consisting of β-amino acid unit, was also stable under neutral conditions, whereas **8,** bearing β, β-dimethyl-β-alanine linker, spontaneously degraded to **1** with a moderate reaction kinetics (*t_1/2_* = 31 min). A large increase of reaction rate was also observed in probe **9,** possessing rigid azabicyclo[2.2.1]heptene linker (*t_1/2_* = 1.2 min) [12]. On the other hand, introduction of cyclopropyl [11] and proline linker resulted in deceleration of the degradation of **10** (*t_1/2_* = 11.6 h) and **11** (*t_1/2_* > 100 h), respectively.

The rapid reaction kinetics of **5** prompted us to evaluate the utility of trimethyl carbamate in spontaneous degradation triggered by other functional groups. We thus prepared a set of coumarin derivatives **12**–**16** and evaluated their auto-cleavage reactions by fluorescence measurement under neutral aqueous conditions (50 mM HEPES buffer, pH 7.4). The results are summarized in Table 2. We found that the amine probe **12** rapidly liberated coumarin **1** (*t_1/2_* = 75 s) while its corresponding *N*-Boc derivative **13** remained stable. Interestingly, the hydroxyl derivatives **14** and **15** decomposed with a moderate reaction kinetics to release **1** (*t_1/2_* = 1.1 and 0.92 h, respectively). On the other hand, the ketone **16** stably existed under neutral aqueous conditions. The results suggested nucleophilic amino and hydroxyl groups of **12**, **14,** and **15** participated in intramolecular cyclization, thereby inducing the spontaneous release of coumarin **1** (Figure 1d).

### 2.2. Fluorescence Detection of Enzyme Reactions with the Trimethyl Carbamate Linker

**Figure 3:**
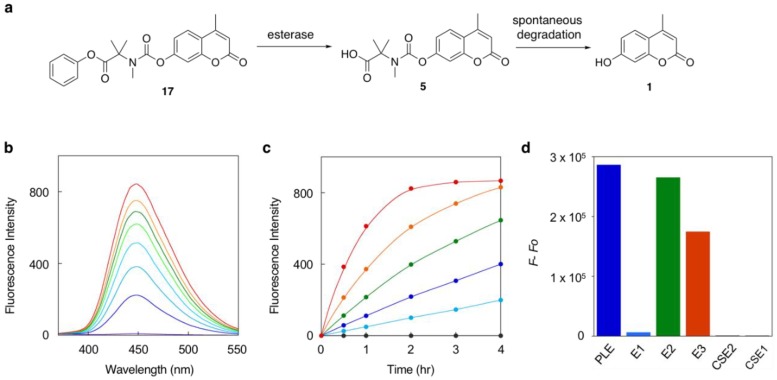
Fluorescence detection of esterase-catalyzed hydrolysis of the probe **17**. (**a**) Sequential reaction of **17,** consisting of esterase-catalyzed hydrolysis and subsequent spontaneous degradation. (**b**) Fluorescence spectral change of **17** upon incubation with PLE (1.0 unit) for 0, 0.5, 1, 1.5, 2, 2.5, 3, and 4 h (from bottom to top). (**c**) Time-trace plots of change in fluorescence intensity (λ_em_ = 450 nm) upon incubation of **17** with 0, 0.1, 0.25, 0.5, 1, and 2 units of PLE (from lower to upper curve). (**d**) Fluorescence intensity change of **17** (λ_em_ = 450 nm) upon incubation with various esterases for 3 h. Measurement conditions: [**17**] = 5 μM, 50 mM HEPES, pH 7.4, 37 °C, λ_ex_ = 320 nm.

With the versatile trimethyl carbamate as self-immolative linker in hand, we next evaluated its utility in fluorescence detection of enzyme reactions. We initially employed the methyl ester **6** for the hydrolysis reaction catalyzed by porcine liver esterase (PLE). However, the fluorescence increase was rather small even after 6 h, suggesting **6** as not-so-suitable substrate of PLE. To facilitate the enzyme reaction, we designed the phenyl ester **17** and evaluated its hydrolysis efficiency (Figure 3a). Results showed the rate of fluorescence increased depending on the concentration of PLE (0–2.0 unit/min) and the emission intensity increased up to >2000-fold at the reaction saturation point (Figure 3b,c). The data together suggested that **17** served as a good fluorogenic substrate of PLE. We further evaluated the hydrolysis efficiency of **17** by other esterases. The data indicated that **17** served as a fluorogenic substrate of esterases E2 and E3, but not of carboxy esterases 1 and 2, and esterase E1 (Figure 3d). This enzyme specificity might be attributable to the unique phenyl ester structure of **17** connected to trimethyl carbamate linker.

**Figure 4:**
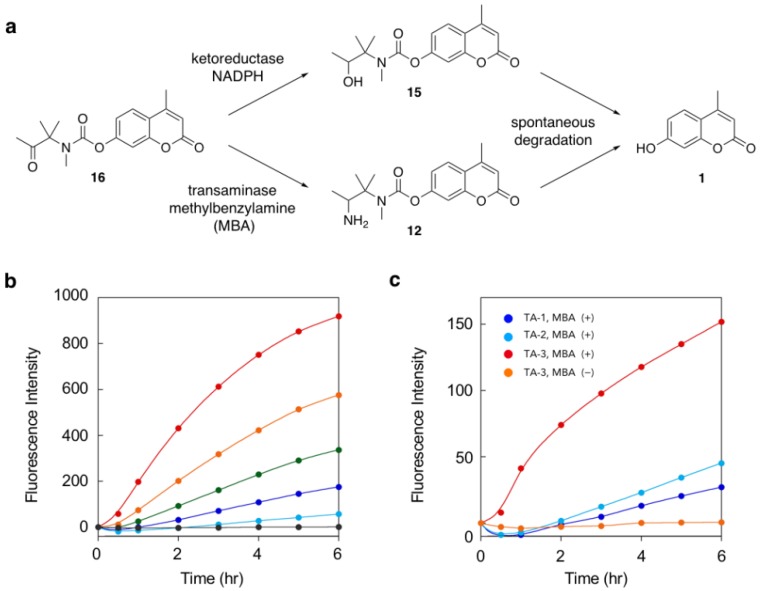
Fluorescence detection of enzyme-catalyzed conversion of keto probe **16**. (**a**) Sequential reactions of **16** to release **1**, induced by ketoreductase or transaminase. (**b**) Time-trace plot of fluorescence intensity change (λ_em_ = 450 nm) upon incubation of **16** with 0.2, 0.5, 1, 2, and 5 mg/mL of ketoreductase K-6 (from lower to upper curve) in presence of NADPH. The lowest black line indicates the result with 5 mg/mL of K-6 in absence of NADPH. Measurement conditions: [**16**] = 10 µM, [NADPH] = 0.2 mg/mL, [glucose] = 10 mg/mL, [glucose dehydrogenase] = 0.2 mg/mL, λ_ex_ = 320 nm, 50 mM phosphate buffer, pH 8.0, 30 °C. (**c**) Time-trace plot of fluorescence intensity change (λ_em_ = 450 nm) upon incubation of **16** with transaminase TA-3 in the presence or absence of methylbenzylamine (MBA). Measurement conditions: [**16**] = 10 µM, (transaminase) = 5 mg/mL, (MBA) = 11.5 µM, (pyridoxal phosphate) = 0.1 µM, λ_ex_ = 320 nm, 100 mM phosphate buffer, pH 7.0, 30 °C.

Utility of the self-immolative linker was further evaluated in fluorescence detection of enzymatic reactions catalyzed by transaminase and ketoreductase (Figure 4a). We found that the fluorescence of keto probe **16** increased upon treatment with ketoreductase K-6 (Figure 4b). The rate of fluorescence increase (λ_em_ = 450 nm) depended on the amount of ketoreductase. Fluorescence change was not observed in the absence of NADPH, which is an enzyme co-factor. These results suggested the reduction of **16** by K-6 to the corresponding alcohol **15**, which spontaneously liberated coumarin **1** to exhibit enhanced fluorescence. We also found that **16** served as a substrate of transaminase (Figure 4c). Treatment of **16** with transaminase TA-3 in presence of methylbenzylamine as a co-substrate induced an increase in fluorescence while removal of methylbenzylamine from the reaction solution completely suppressed the increase. Other transaminases TA-1 and TA-2 induced slight fluorescence enhancements. These data suggested that TA-3 efficiently catalyzed the conversion of **16** to the corresponding amine **12**, which spontaneously decomposed to fluorescent coumarin **1**.

**Figure 5:**
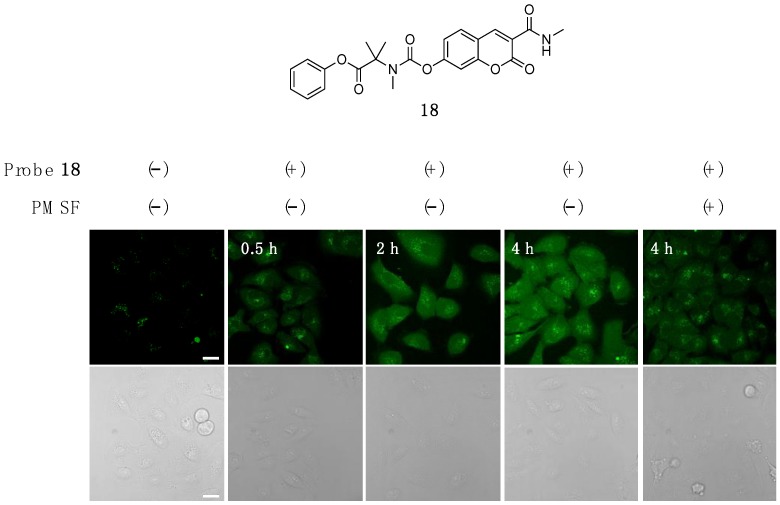
Fluorescence detection of intracellular esterase activity in living A549 cells using **18**. Cells were incubated with **18** at the indicated time at 37 °C in the presence or absence of PMSF. Conditions: (**18**) = 10 μM, (PMSF) = 500 μM, HBS buffer, λ_ex_ = 405 nm. Scale bar: 20 μm.

Finally, we further evaluated the utility of trimethyl carbamate in the fluorescence imaging of esterase activity in living cells. We prepared 3-carboxyamide coumarin probe **18**, which was able to be excited with blue laser (λ_ex_ = 405 nm) for confocal fluorescence microscopy analysis (Figure 5). Cell viability assay revealed that probe **18** did not exert significant cells toxicity at 10 μM (Figure S1). We also confirmed that **18** was stable in the presence of physiologically relevant concentration of glutathione (5 mM) under the neutral conditions (Figure S2). When A549 cells were incubated with **18** (10 μM in HBS) at 37 °C, fluorescence due to coumarin gradually increased in a time-dependent manner in the cytosol of cells. The fluorescence intensity apparently decreased at 4 h when the cells were pre-treated with phenylmethylsulfonyl fluoride (PMSF) as a pan-esterase inhibitor. The results together suggested that **18** was hydrolyzed by intracellular esterase(s) to liberate the fluorescent coumarin **1** inside the cells.

## 3. Materials and Methods 

### 3.1. Fluorescence Measurement and Reaction Kinetics Analysis of the Coumarin Probes

The fluorescence spectrum was measured by a PerkinElmer LS-55 spectrofluorophotometer. A quartz cell containing the coumarin probe (5 μM) in HEPES buffer (50 mM, pH = 7.4) was placed at the cell holder and measured the fluorescence (λ_ex_ = 320 nm) by spectral mode or time-drive mode. For reaction kinetics analysis, plot of the fluorescence intensity at 450 nm was analyzed by nonlinear least squares curve-fitting as a first order reaction to obtain rate constant (*k*, h^−1^) and *t_1/2_* (h). 

### 3.2. Fluorescence Detection of Esterase Activity Using Probe 17

A HEPES buffer (50 mM, pH = 7.4) containing probe **17** (10 μM) and esterase was incubated at 37 °C. The fluorescence spectrum was measured at each reaction time point using PerkinElmer LS-55 spectrofluorophotometer (λ_ex_ = 320 nm). 

### 3.3. Fluorescence Detection of Ketoreductase Activity Using Probe 16

A phosphate buffer (50 mM, pH = 8.0) containing probe **16** (10 μM), ketoreductase (5 mg/mL, kindly provided from Amano Enzyme Inc., Aichi, Japan), NADPH (0.2 mg/mL), glucose dehydrogenase (0.2 mg/mL) and glucose (10 mg/mL) was incubated at 30 °C. The fluorescence intensity of the solution (λ_em_ = 450 nm) was measured at each time point by PerkinElmer EnSpire Multimode Plate Reader (λ_ex_ = 320 nm).

### 3.4. Fluorescence Detection of Transaminase Activity Using Probe 16

A phosphate buffer (100 mM, pH = 7.0) containing probe **16** (10 μM), transaminase (5 mg/mL, kindly provided from Amano Enzyme Inc.), methylbenzylamine (11.5 µM), and pyridoxal phosphate (100 µM) was incubated at 30 °C. The fluorescence intensity of the solution (λ_em_ = 450 nm) was measured at each reaction time point using PerkinElmer EnSpire Multimode Plate Reader (λ_ex_ = 320 nm).

### 3.5. Cell Culture

A549 cells were cultured in high-glucose Dulbecco’s Modified Eagle medium (DMEM, 4.5 g of glucose/L, Sigma-Aldrich, St. Louis, United States) supplemented with 10% fetal bovine serum (FBS), penicillin (100 units/mL) and streptomycin (100 μg/mL) under humidified atmosphere of 5% CO_2_ in air. Subculture was performed every 3–4 days from subconfluent (~80%) cultures using trypsin-EDTA solution.

### 3.6. Evaluation of Cell Viability after Treatment of A549 Cells with Probe 18

A549 cells seeded on 3.5 cm dish (Falcon) were cultured for 2 days at 37 °C in CO_2_ incubator. The A549 cells were treated with or without probe **18** (10 μM) for 3 h at 37 °C in HBS(+) buffer (20 mM HEPES, 107 mM NaCl, 6 mM KCl, 1.2 mM MgSO_4_, 2 mM CaCl_2_, 11.5 mM glucose, adjusted to pH 7.4 with NaOH). The cell viability was evaluated by the standard typan blue assay. Error bars represent standard deviation from the mean (*n* = 3).

### 3.7. Evaluation of Reaction of 18 and Glutathione

A degassed HEPES buffer (50 mM, pH = 7.4) containing probe **18** (10 μM), TCEP (10 mM) and glutathione (5 mM) was incubated at 37 °C for 4 h. The fluorescence spectrum was measured using PerkinElmer LS-55 spectrofluorophotometer (λ_ex_ = 405 nm).

### 3.8. Fluorescence Imaging of Intracellular Esterases in Living A549 Cells

A549 cells seeded on 3.5 cm glass-base dish (Iwaki) were cultured for 2–3 days at 37 °C in CO_2_ incubator. The cells were washed with HBS (+) buffer twice and treated with probe 18 for 4 h at 37 °C in HBS (+) buffer. In control experiment, the cells were pre-treated with PMSF (500 μM) for 2 h at 37 °C in HBS (+) buffer, and then treated with probe **18** as described above in the presence of PMSF (500 μM). The cells were subjected to fluorescence imaging without wash. Fluorescence imaging was performed with confocal microscopy (TCS SP8, Leica microsystems) equipped with HyD detector (405 nm excitation derived from a semiconductor laser).

## 4. Conclusions

We have reported the development of trimethyl carbamate as a new class of self-immolative linker. The rapid-to-moderate reaction kinetics of trimethyl carbamate, in response to the enzyme-catalyzed structural change, while the high chemical stability of the parent ester and keto probe, allowed us to detect the activities of different enzymes, including esterase, ketoreductase, and transaminase. We believe that, as a versatile building block, trimethyl carbamate could widen the choice of available self-immolative linkers in fluorescent probe design, and thereby contribute to development of fluorescence-based biological analyses. Not only in enzyme activity analysis, trimethyl carbamate could also serve as a functional scaffold in stimuli-responsive materials and drug delivery systems.

## Figures and Tables

**Table 1:**
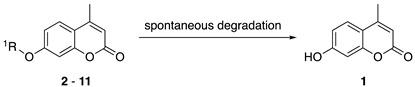
Summary of the reaction kinetics parameters of coumarin carbamate probes bearing different linker units ^a^.

^1^R	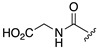	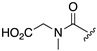	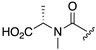	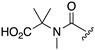	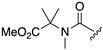
	**2**	**3**	**4**	**5**	**6**
*k* (h^−1^)	n.d. ^b^	0.023	1.2	120	n.d. ^c^
*t_1/2_* (h)	n.d. ^b^	30	0.58 (35 min)	0.0058 (21 s)	> 100 h
^1^R	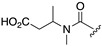	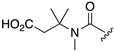	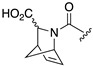	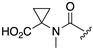	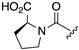
	**7**	**8**	**9**	**10**	**11**
*k* (h^−1^)	n.d. ^c^	1.3	36	0.060	n.d. ^c^
*t_1/2_* (h)	> 100 h	0.53 (32 min)	0.019 (1.2 min)	11.6	> 100 h

^a^ Reaction was monitored by the fluorescence spectral change of coumarin probe. Measurement conditions: [probe] = 5 μM, 50 mM HEPES, pH 7.4, 37 °C, λ_ex_ = 320 nm. ^b^ Not determined due to instability of the probe under measurement conditions. ^c^ Not determined.

**Table 2:**
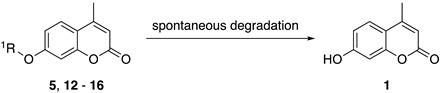
Summary of the reaction kinetics parameters of trimethyl carbamate probes possessing different functional groups ^a^.

^1^R	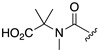	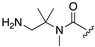	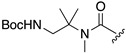
	**5**	**12**	**13**
*k* (h^−1^)	120	92	n.d. ^b^
*t_1/2_* (h)	0.0058 (21 s)	0.021 (75 s)	> 100 h
^1^R	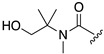	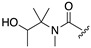	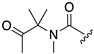
	**14**	**15**	**16**
*k* (h^−1^)	0.64	0.75	n.d.^b^
*t_1/2_* (h)	1.1 h	0.92 h	> 100 h

^a^ Reaction was monitored by fluorescence spectral change of coumarin probe. Measurement conditions: [probe] = 5 μM, 50 mM HEPES, pH 7.4, 37 °C, λ_ex_ = 320 nm. ^b^ Not determined.

## Supplementary Materials

The following are available online, detail of synthesis of compounds. Figure S1. Evaluation of cell viability after treatment of A549 cells with probe **18**, Figure S2. Evaluation of stability of coumarin probe **18** in the presence of glutathione.
