# Supplementary material for: Trimethyl-Substituted Carbamate as a Versatile Self-Immolative Linker for Fluorescence Detection of Enzyme Reactions
Source: Molecules. 2020 May 5;25(9):2153. doi: 10.3390/molecules25092153 (PMC7249185; doi:10.3390/molecules25092153)
Supplement: Supplementary file 1 [file molecules-25-02153-s001.pdf]

Supporting Information for

Trimethyl-Substituted Carbamate as a Versatile Self-Immolative Linker for Fluorescence Detection of Enzyme Reactions

Noriaki Nakamura, Shohei Uchinomiya, Kazuya Inoue, Akio Ojida*

Graduate School of Pharmaceutical Sciences, Kyushu University, Fukuoka, Japan

Table of Contents

Figure S1. Evaluation of cell viability after treatment of A549 cells with probe **18**.

Figure S2. Evaluation of stability of coumarin probe **18** in the presence of glutathione.

Experimental Details

Synthesis and Characterization of the Compounds

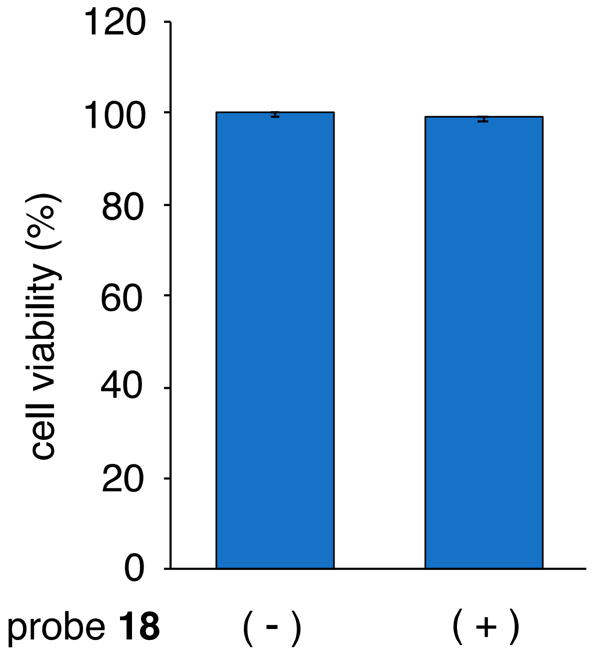

Figure S1. Evaluation of cell viability upon treatment of A549 cells with probe **18**. A549 cells were treated with probe **18** (10 μM) for 3 h at 37 °C in HBS buffer (pH 7.4). The test was conducted by standard typan blue assay. Error bars represent standard deviation from the mean (n = 3).

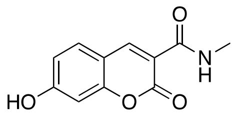

18-2

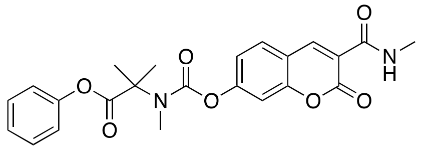

18

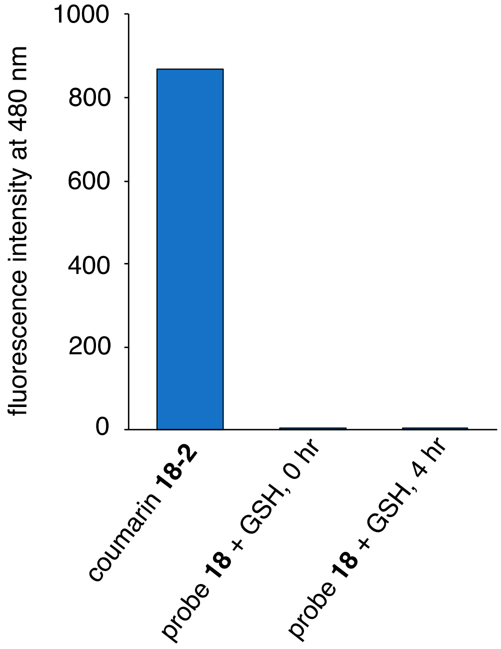

Figure S2. Evaluation of stability of coumarin probe **18** in the presence of glutathione (GSH). A solution of **18** (10 μM) was incubated for 4h in degassed HEPES buffer (50 mM, pH = 7.4) at 37 °C in the presence of GSH (5 mM) and TCEP (10 mM). The fluorescence intensity at 480 nm was measured (λ_{ex} = 405 nm). **18** showed negligible fluorescence after 4 h as compared to that of the parent coumarin **18-2** (10 μM), suggesting that **18** stably existed in the presence of the physiologically relevant concentration GSH.

General materials and methods for organic synthesis

Unless otherwise noted, chemical reagents were purchased from commercial suppliers (FUJIFILM Wako Pure Chemical Corporation, Tokyo Chemical Industry, Sigma-Aldrich, Watanabe Chemical Industries) and used without further purification. Reactions were carried out under a positive atmosphere of nitrogen, unless otherwise stated. Reactions were monitored by thin layer chromatography (TLC) carried out on Merck TLC Silica gel 60 F_{254}, using shortwave UV light as the visualizing agent. ^{1}H NMR spectra were recorded using a Varian UNITY-400 (400 MHz) spectrometer or Bruker Avance III HD 500 MHz spectrometer and chemical shifts (δ, ppm) were referenced to residual solvent peak ($CDCl_3$: 7.26 ppm; MeOH-d_4: 3.31 ppm; DMSO-d_6: 2.50 ppm). ESI mass spectrometry was recorded using a MicroTOF II (Bruker Daltonics) spectrometer. HPLC purification was conducted with a HITACHI L-7000 series (Hitachi).

Preparation of **2**

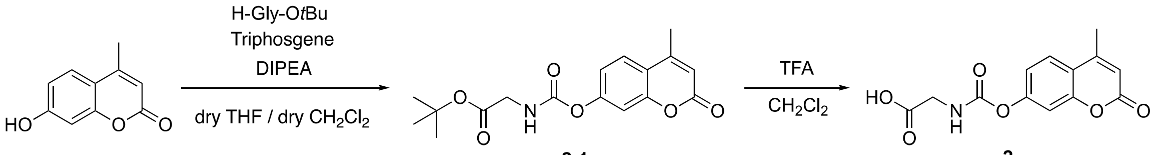

1 **2-1** **2**

Synthesis of **2-1**

To a solution of 7-hydroxy-4-methylcoumarin **1**[S1] (50.0 mg, 0.284 mmol) and DIPEA (49.5 µL, 0.284 mmol) in dry THF (2.5 mL) was added triphosgen (28.7 mg, 96.7 µmol) in dry DCM (3 mL) and the mixture was stirred for 1h at room temperature. To the solution were added Glycine tert-Butyl Ester Hydrochloride (71.4 mg, 0.426 mmol) and DIPEA (74.2 µl, 0.426 mmol) in dry DCM (2.5 mL) and the mixture was stirred for 30 min at room temperature. The solution was diluted with chloroform and washed with sat. $NaHCO_3$ aq., water and brine. After removal of the solvent by evaporation, the residue was purified by column chromatography on SiO_2 (hexane : ethyl acetate = 5 : 1 → 2 : 1) to give **2-1** (34.0 mg, 36%) as a white solid. ^{1}H-NMR (500 MHz, $CDCl_3$) : δ 1.50 (9H, s), 2.42 (3H, s), 3.96-3.97 (2H, d, *J* = 5.5 Hz), 5.60-5.62 (1H, t, *J* = 5.0 Hz), 6.25 (1H, d, *J* = 1.5 Hz), 7.12-7.14 (1H, dd, *J* = 1.5 Hz, 9.0 Hz), 7.16 (1H, d, *J* = 2.5 Hz), 7.57-7.58 (1H, d, *J* = 8.5 Hz). ESI-TOF-MS: m/z for $C_{17}H_{19}NO_6Na$ $[M+Na]^+$: calcd 356.1105, observed 356.1099.

Synthesis of **2**

To a solution of **2-1** (17.0 mg, 50.9 µmol) in dry DCM (3.0 mL) was added TFA (0.3 mL) and the mixture was stirred for 11 hr at room temperature. The solvent was removed by evaporation to give **2** (18.0 mg, quant) as a white solid. **2** was used for evaluating the reaction kinetics of intramolecular cyclization without further purification. ^{1}H-NMR (500 MHz, CD_3OD) : δ 2.49 (3H, d, *J* = 1.5 Hz), 3.93 (2H, s), 5.49 (1H, s), 6.30-6.31 (1H, d, *J* = 1.5 Hz), 7.19-7.22 (2H, m), 7.78-7.80 (1H, d, *J* = 8.5 Hz). ESI-TOF-MS: m/z for $C_{13}H_{11}NO_6Na$ $[M+Na]^+$: calcd 300.0479, observed 300.0495.

Preparation of **3**

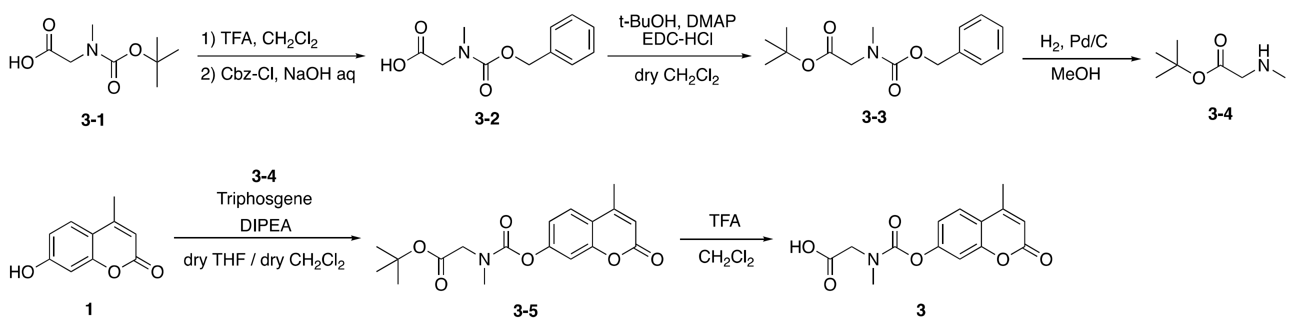

Synthesis of **3-2**

To a solution of **3-1** (500 mg, 2.64 mmol) in dry DCM (10 mL) was added TFA (2.0 mL) and the mixture was stirred for 1.5 hr at room temperature. After removal of the solvent by evaporation, the residue was diluted in 2N NaOH aq. (10 mL). To the solution was added benzyl chloroformate (1.13 mL, 7.92 mmol) and the mixture was stirred for 3h at 0°C. The solution was diluted with water and the aqueous layer was washed with ether. After adjustment of pH of the aqueous layer to 1 by 5 N HCl aq., the product was extracted with ethyl acetate. The organic layer was dried over Na_2SO_4 to give **3-2** (749 mg, quant) as colorless oil. ^{1}H-NMR (500 MHz, DMSO-d_6) : δ 2.88-2.92 (3H, d, J = 21.0 Hz), 3.94-3.97 (2H, d, J = 16.5 Hz), 5.05-5.09 (2H, d, J = 19.5 Hz), 7.30-7.38 (5H, m), 12.68 (1H, brs). ESI-TOF-MS: m/z for $C_{11}H_{13}NO_4Na$ $[M+Na]^+$: calcd 246.0742, observed 246.0752.

Synthesis of **3-3**

To a solution of **3-2** (180 mg, 0.806 mmol), EDC-HCl (171 mg, 0.887 mmol), DIPEA (211 µL, 1.21 mmol) and DMAP (29.6 mg, 0.242 mmol) in dry DCM (2.0 mL) was added *tert*-BuOH (115 µL, 1.21 mmol) and the mixture was stirred for 4.5 hr at room temperature. The mixture was diluted with ethyl acetate and the organic layer was washed with sat. $NaHCO_3$ aq., water and brine. After removal of the solvent by evaporation, the residue was purified by column chromatography on SiO_2 (hexane : ethyl acetate = 5 : 1) to give **3-3** (98.3 mg, 43%) as colorless oil. ^{1}H-NMR (400 MHz, $CDCl_3$) : δ 1.41-1.47 (9H, d, J = 21.2 Hz), 2.99-3.00 (3H, d, J = 4.4 Hz), 3.87-3.94 (2H, d, J = 27.6 Hz), 5.13-5.15 (2H, d, J = 10 Hz), 7.32-7.36 (5H, m). ESI-TOF-MS: m/z for $C_{15}H_{21}NO_4Na$ $[M+Na]^+$: calcd 302.1363, observed 302.1377.

Synthesis of **3-4**

To a solution of **3-3** (98.3 mg, 0.352 mmol) in MeOH (6.0 mL) was added Pd/C (24.0 mg) and the mixture was stirred under H_2 atmosphere for 3hr at room temperature. After filtration thorough celite, the solvent was removed by evaporation to give **3-4** (33.2 mg, 65%) as white solid and used next reaction without further purification. ^{1}H-NMR (400 MHz, $CDCl_3$) : δ1.50 (9H, s), 2.78 (3H, s), 3.63 (2H, s). ESI-TOF-MS: m/z for $C_7H_{16}NO_2$ $[M+H]^+$: calcd 146.1181, observed 146.1190.

Synthesis of **3-5**

To a solution of **1** (26.4 mg, 0.150 mmol) and DIPEA (26.1 μl, 0.150 mmol) in dry THF (1.0 mL) was added triphosgene (14.8 mg, 50.0 μmol) in dry DCM (1.5 mL) by dropwise and the mixture was stirred for 1hr at room temperature. To the solution were added **3-4** (32.7 mg, 0.225 mmol) and DIPEA (39.2 μl, 0.225 mmol) and the mixture was stirred for 1hr at room temperature. The mixture was diluted with chloroform and the organic layer was washed with sat. $NaHCO_3$ aq., water and brine. After removal of the solvent by evaporation, the residue was purified by column chromatography on SiO_2 (hexane : ethyl acetate = 2 : 1) to give **3-5** (18.6 mg, 36%) as awhite solid. ^{1}H-NMR (500 MHz, $CDCl_3$) : δ 1.49-1.50 (9H, s), 2.42 (3H, s), 3.07-3.17 (3H, d, *J* = 47.5 Hz), 4.00-4.03 (2H, d, *J* = 14 Hz), 6.25 (1H, s), 7.09-7.16 (2H, m), 7.57-7.59 (1H, dd, *J* = 2.5 Hz, 9.5 Hz). ESI-TOF-MS: m/z for $C_{18}H_{21}NO_6Na$ $[M+Na]^+$: calcd 370.1261, observed 370.1273.

Synthesis of **3**

To a solution of **2-5** (9.3 mg, 26.8 μmol) in dry DCM (2.0 mL) was added TFA (0.4 mL) and the mixture was stirred for 2hr at room temperature. The solvent was removed by evaporation to give **2** (7.6 mg, 96%) as a white solid. **3** was used for evaluating the reaction kinetics of intramolecular cyclization without further purification. ^{1}H-NMR (500 MHz, CD_3OD) : δ2.48 (3H, s), 3.13 (3H, d, *J* = 64.5 Hz), 4.15 (2H, d, *J* = 50.5 Hz), 6.31 (1H, s), 7.13-7.15 (1H, m), 7.19-7.22 (1H, m), 7.79 (1H, d, *J* = 8.0 Hz). ESI-TOF-MS: m/z for $C_{18}H_{15}NO_6Na$ $[M+Na]^+$: calcd 314.0641, observed 314.0642.

Preparation of **4**

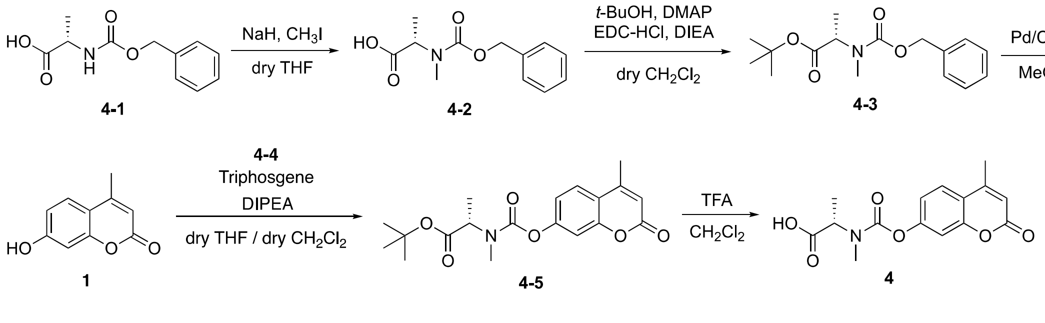

Synthesis of **4-2**

To a solution of **4-1** (500 mg, 2.24 mmol) and NaH (270 mg, 6.72 mmol) in dry THF (5.0 mL) was added CH_3I (791 μL, 12.7 mmol) at 0°C and the mixture was stirred for 7 hr at room temperature. The reaction was quenched by addition of water and the aqueous layer was washed with ethyl acetate. After adjustment of pH to 2 by 1N HCl aq., the product was extracted with ethyl acetate. The solvent was removed by evaporation to give **4-2** (540 mg, quant) as yellow oil. ^{1}H-NMR (500 MHz, DMSO-d_6) : δ 1.32-1.34 (3H, dd, *J* = 6.0 Hz, 7.5 Hz), 2.81-2.83 (3H, d, *J* = 7.0 Hz), 4.53-4.62 (1H, dq, *J* = 7.5 Hz, 25 Hz), 5.06-5.09 (2H, d, , *J* = 16 Hz), 7.32-7.37 (5H, m), 12.69 (1H, brs). ESI-TOF-MS: m/z for $C_{12}H_{15}NO_4Na$ $[M+Na]^+$: calcd 260.0893, observed 260.0821.

Synthesis of **4-3**

To a solution of **4-2** (540 mg, 2.27 mmol), EDC-HCl (479 mg, 2.50 mmol), DIPEA (593 μL, 3.41 mmol) and

DMAP (83.2 mg, 0.681 mmol) in dry DCM (8.0 mL) was added *tert*-BuOH (323 µL, 3.40 mmol) and the mixture was stirred for 6.5 hr at room temperature. The mixture was diluted with chloroform and the organic layer was washed with sat. $NaHCO_3$ aq., water and brine. After removal of the solvent by evaporation, the residue was purified by column chromatography on SiO_2 (hexane : ethyl acetate = 5 : 1) to give **4-3** (370 mg, 55%) as colorless oil. ^{1}H-NMR (500 MHz, $CDCl_3$) : δ 1.36-1.38 (3H, d, *J* = 7.5 Hz), 1.40-1.44 (3H, d, *J* = 19.5 Hz), 2.87-2.90 (3H, d, *J* = 14.5 Hz), 4.58-4.81 (1H, dq, *J* = 7 Hz, 97.5 Hz), 5.08-5.22 (2H, m), 7.29-7.36 (5H, m). ESI-TOF-MS: m/z for $C_{16}H_{23}NO_4Na$ $[M+Na]^+$: calcd 316.1519, observed 316.1515.

Synthesis of **4-4**

To a solution of **4-3** (370 mg, 1.26 mmol) in MeOH (15.0 mL) was added Pd/C (80.0 mg) and the mixture was stirred for 7.5 hr at room temperature under H_2 atmosphere. After filtration thorough celite, the solvent was removed by evaporation to give **4-4** (122 mg, 61%) as white solid and used next reaction without further purification. ^{1}H-NMR (500 MHz, $CDCl_3$) : δ 1.22-1.23 (3H, d, *J* = 7.0 Hz), 1.45 (9H, s), 2.35 (3H, s), 3.07-3.12 (1H, q, *J* = 7.0 Hz). ESI-TOF-MS: m/z for $C_8H_{17}NO_2$ $[M+H]^+$: calcd 160.1332, observed 160.1338.

Synthesis of **4-5**

To a solution of **1** (46.4 mg, 0.263 mmol) and DIPEA (45.9 µL, 0.263 mmol) in dry THF (2.0 mL) was added triphosgene (26.1 mg, 87.8 µmol) in dry DCM (2.0 mL) by dropwise and the mixture was stirred for 2hr at room temperature. To the solution were added **4-4** (62.8 mg, 0.395 mmol) and DIPEA (68.8 µL, 0.395 mmol) in dry DCM (2.0 mL) by dropwise and the mixture was stirred for 1hr at room temperature. The mixture was diluted with chloroform and the organic layer was washed with sat. $NaHCO_3$ aq., water and brine. After removal of the solvent by evaporation, the residue was purified by column chromatography on SiO_2 (chloroform) to give **4-5** (46.2 mg, 49%) as colorless oil. ^{1}H-NMR (500 MHz, $CDCl_3$) : δ 1.43-1.49 (3H, dd, *J* = 8.5 Hz, 24.5 Hz), 1.46 (9H, s), 2.40 (3H, d, *J* = 1.0 Hz), 2.96-3.04 (3H, d, *J* = 39 Hz), 4.66-4.78 (1H, dq, *J* = 8.5 Hz, 36 Hz), 6.21 (1H, d, *J* = 1.0 Hz), 7.05-7.12 (2H, m), 7.55-7.57 (1H, d, *J* = 9.0 Hz). ESI-TOF-MS: m/z for $C_{19}H_{23}NO_6Na$ $[M+Na]^+$: calcd 384.1418, observed 384.1405.

Synthesis of **4**

To a solution of **4-5** (6.2 mg, 17.2 µmol) in dry DCM (2.0 mL) was added TFA (0.4 mL) and the mixture was stirred for 3 hr at room temperature. The solvent was removed by evaporation to give **4** (8.0 mg, quant) as a white solid. **4** was used for evaluating the reaction kinetics of intramolecular cyclization without further purification. ^{1}H-NMR (500 MHz, CD_3OD) : δ 1.51-1.58 (3H, dd, *J* = 27.0 Hz), 2.49 (3H, s), 3.01-3.11 (3H, d, *J* = 51.0 Hz), 4.75-4.85 (1H, m), 6.31 (1H, s), 7.14-7.21 (2H, m), 7.78-7.81 (1H, m). ESI-TOF-MS: m/z for $C_{15}H_{15}NO_6$ $[M+H]^+$: calcd 328.0792, observed 328.0766.

Preparation of **5**

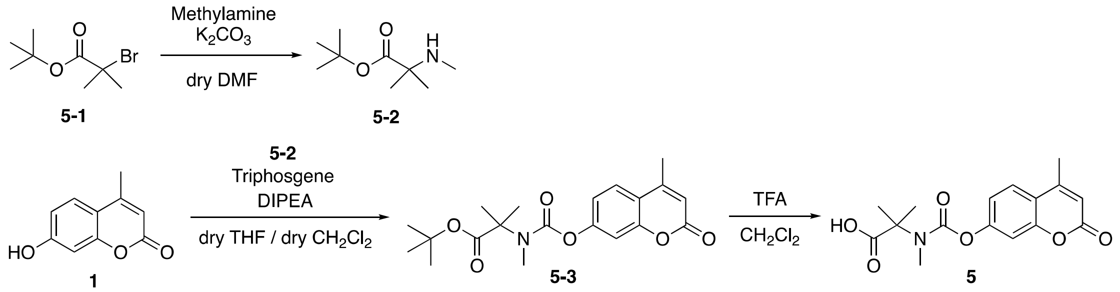

Synthesis of **5-2**

To a solution of **5-1** (300 mg, 1.34 mmol) and K_2CO_3 (556 mg, 4.02 mmol) in dry DMF (4.5 mL) was added methylamine (157 mg, 2.02 mmol) and the mixture was stirred for 18.5 hr at 60°C. After dilution with 1N citric acid aq., the aqueous layer was washed with ethyl acetate. After adjustment of pH of the solution to 12 by 4N NaOH aq., the product was extracted with ether. The solvent was removed by evaporation to give **5-2** (180 mg, 78%) as colorless oil. ^{1}H-NMR (500 MHz, DMSO-d_6) : δ 1.12 (6H, s), 1.40 (9H, s), 2.13 (3H, s). ESI-TOF-MS: m/z for $C_9H_{19}NO_2$: calcd 174.1489 $[M+H]^+$, observed 174.1502.

Synthesis of **5-3**

To a solution of **1** (34.5 mg, 0.196 mmol) and DIPEA (34.1 µL, 0.196 mmol) in dry THF (1.5 mL) was added triphosgene (19.4 mg, 65.3 µmol) in dry DCM (1.5 mL) by dropwise and the mixture was stirred for 2 hr at room temperature. To the solution were added **5-2** (84.8 mg, 0.490 mmol) and DIPEA (85.3 µL, 0.490 mmol) in dry DCM (2.0 mL) by dropwise and the mixture was stirred for 1.5 hr at room temperature. The mixture was diluted with chloroform and the organic layer was washed with sat. $NaHCO_3$ aq., water and brine. After removal of the solvent by evaporation, the residue was purified by column chromatography on SiO_2 (hexane : ethyl acetate : NH_3 aq.= 400 : 200 : 1) to give **5** (16.8 mg, 23%) as colorless oil. ^{1}H-NMR (500 MHz, $CDCl_3$) : δ 1.44 (9H, s), 1.50 (6H, s), 2.41 (3H, s), 3.11 (3H, s), 6.23 (1H, s), 7.08-7.10 (1H, dd, J = 2.5 Hz, 8.5 Hz), 7.11 (1H, s), 7.55-7.57 (1H, d, J = 8.5 Hz). ESI-TOF-MS: m/z for $C_{20}H_{25}NO_6Na$ $[M+Na]^+$: calcd 398.1574, observed 398.1600.

Synthesis of **5**

To a solution of **5-3** (5.7 mg, 15.2 µmol) in dry DCM (2.0 mL) was added TFA (0.4 mL) and the mixture was stirred for 2.5 hr at room temperature. The solvent was removed by evaporation to give **5** (6.2 mg, quant) as a white solid. **5** was used for evaluating the reaction kinetics of intramolecular cyclization without further purification. Measurement of NMR spectrum of **5** was not conducted due to rapid intramolecular cyclization of **5**. ESI-TOF-MS: m/z for $C_{16}H_{17}NNaO_6Na$ $[M+Na]^+$: calcd 342.0954 , observed 342.0887.

Preparation of **6**

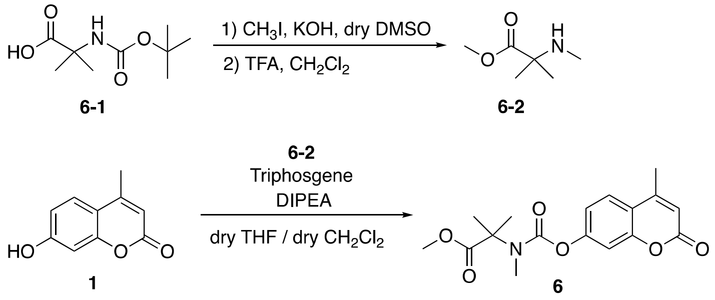

Synthesis of **6-2**

To a solution of **6-1** (500 mg, 2.46 mmol) and KOH (690 mg, 12.30 mmol) in dry DMSO (5 mL) was added CH_3I (615 µl, 9.84 mmol) and the mixture was stirred for 1 hr at room temperature. The reaction was quenched by addition of water and the mixture was extracted with ether. After removal of the solvent by evaporation, the residue was diluted with dichloromethane (10 mL) and TFA (4 mL) and stirred for 1hr at room temperature. After removal of the solvent by evaporation, the residue was diluted with water and washed with ethyl acetate. After adjustment of pH to 12 with 1N NaOH aq., the product was extracted with ether to give **6-2** (274 mg, quant) as colorless oil. ^{1}H-NMR (500 MHz, $CDCl_3$) : δ1.28 (6H, s), 2.23 (3H, s), 3.45 (1H, s), 3.70 (3H, s) . ESI-TOF-MS: m/z for $C_6H_{14}NO_2Na$ $[M+Na]^+$: calcd 132.1025, observed 132.1007.

Synthesis of **6**

To a solution of **1** (27.2 mg, 0.150 mmol) and DIPEA (27.2 µL, 0.150 mmol) in dry THF (1.0 mL) was added triphosgene (16.7 mg, 0.0500 mmol) in dry DCM (1.0 mL) by dropwise and the mixture was stirred for 1.5 hr at room temperature. To the solution were added **6-2** (30.0 mg, 0.220 mmol) and DIPEA (40.8 µl, 0.220 mmol) in dry DCM (1.5 mL) by dropwise and the mixture was stirred for 1.5 hr at room temperature. The mixture was diluted with chloroform and the organic layer was washed with sat. $NaHCO_3$ aq., water and brine. After removal of the solvent by evaporation, the residue was purified by column chromatography on SiO_2 (hexane : ethyl acetate : NH_3 aq.= 400 : 200 : 1 → chloroform : NH_3 aq.= 300 : 1) to give **6** (13.7 mg, 27%) as colorless oil. ^{1}H-NMR (500 MHz, $CDCl_3$) : δ 1.55 (6H, s), 2.41 (3H, s), 3.13 (3H, s), 3.71 (3H, s), 6.23 (1H, s), 7.07-7.10 (2H, m), 7.55-7.57 (1H, d, *J*=8.8 Hz). ESI-TOF-MS: m/z for $C_{17}H_{19}NO_6Na$ $[M+Na]^+$: calcd 356.1110, observed 356.1092.

Preparation of **7**

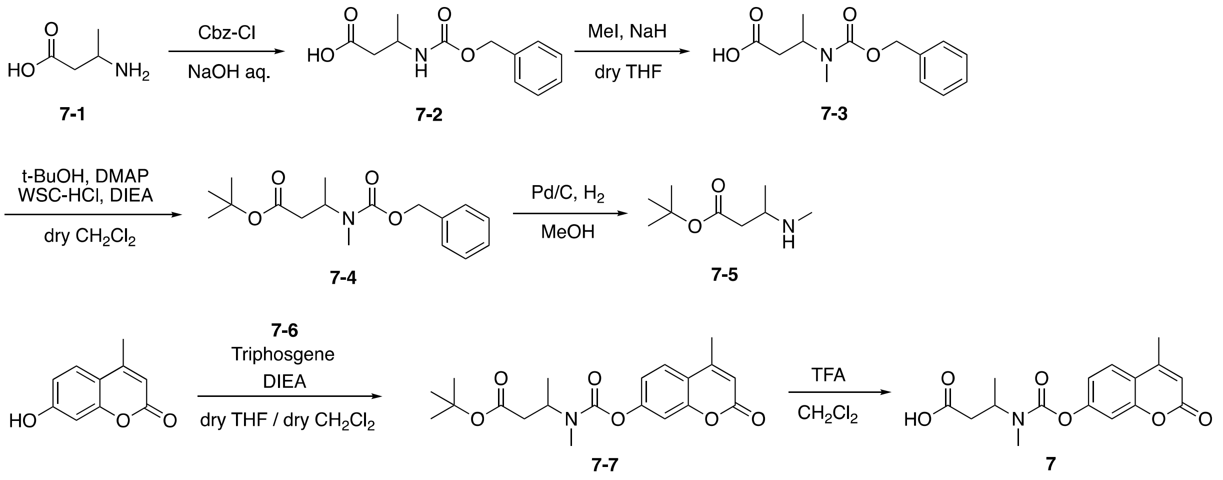

Synthesis of **7-2**

To a solution of **7-1** (300 mg, 2.91 mmol) in 2 N NaOH aq. (10 mL) was added benzyl chloroformate (1.25 mL, 8.73 mmol) and the mixture was stirred for 5.5 hr at 0°C. The mixture was diluted with water and the aqueous layer was washed with ether. After adjustment of pH to 1 by 5 N HCl aq., the product was extracted with ethyl acetate. The solvent was removed by evaporation to give **7-2** (498 mg, 72%) as a white solid. ^{1}H-NMR (500 MHz, DMSO-d$_6$) : δ1.07-1.09 (3H, d, *J* = 8.5 Hz), 2.24-2.45 (2H, m), 3.83-3.89 (1H, dt, *J* = 14 Hz, 7 Hz), 5.00 (2H, s), 7.22-7.35 (5H, m). ESI-TOF-MS: m/z for $C_{12}H_{15}NO_4Na$ [M+Na]$^+$: calcd 260.0899, observed 260.0900.

Synthesis of **7-3**

To a solution of **7-2** (500 mg, 2.11 mmol) and NaH (253 mg, 6.32 mmol) in dry THF (8.0 mL) was added CH_3I (2.62 ml, 42.2 mmol) at 0°C and the mixture was stirred for 12 hr at room temperature. The reaction was quenched by addition of water and the aqueous layer was washed with ethyl acetate. After adjustment of pH to 2 with 5N citric acid aq., the product was extracted with ethyl acetate. The solution was removed by evaporation to give **7-3** (408 mg, 77%) as a yellow oil. ^{1}H-NMR (400 MHz, DMSO-d$_6$) : δ1.10-1.12 (3H, d, *J* = 6.4 Hz), 2.41-2.47 (2H, m), 2.74 (3H, s), 4.47 (1H, s), 5.07 (2H, s), 7.31-7.39 (5H, m), 12.17 (1H, s). ESI-TOF-MS: m/z for $C_{13}H_{17}NO_4Na$ [M+Na]$^+$: calcd 274.1055, observed 274.1063.

Synthesis of **7-4**

To a solution of **7-3** (408 mg, 1.62 mmol), EDC-HCl (342 mg, 1.78 mmol), DIPEA (423 μL, 2.43 mmol) and DMAP (59.4 mg, 0.486 mmol) in dry DCM (5.0 mL) was added *tert*-BuOH (231 μL, 2.43 mmol) and the mixture was stirred for 19 hr at room temperature. The mixture was diluted with chloroform and the organic layer was washed with sat. $NaHCO_3$ aq., water and brine. After removal of the solvent by evaporation, the residue was purified by column chromatography on SiO_2 (hexane : ethyl acetate = 5 : 1) to give **7-4** (253 mg, 51%) as colorless oil. ^{1}H-NMR (500 MHz, $CDCl_3$) : δ1.17 (3H, s), 1.40 (9H, s), 2.33-2.49 (2H, m), 2.80 (3H, s), 4.60-4.67 (1H, m), 5.12 (2H, s), 7.30-7.36 (5H, m). ESI-TOF-MS: m/z for $C_{17}H_{25}NO_4Na$ [M+Na]$^+$: calcd 330.1681, observed 360.1675.

Synthesis of **7-5**

To a solution of **7-4** (253 mg, 0.821 mmol) in MeOH (10 mL) was added Pd/C (50.0 mg) and the mixture was stirred for 3.5 hr at room temperature under H_2 atmosphere. After filtration thorough celite, the solvent was removed by evaporation to give **7-5** (153 mg, quant) as white solid and used next reaction without further purification. ^{1}H-NMR (500 MHz, $CDCl_3$) : δ1.11-1.12 (3H, d, *J* = 6.5 Hz), 1.44 (9H, s), 2.24-2.45 (5H, m), 2.96-3.00 (1H, m). ESI-TOF-MS: m/z for $C_9H_{19}NO_2Na$ $[M+Na]^+$: calcd 196.1313, observed 196.1327.

Synthesis of **7-6**

To a solution of **1** (30.0 mg, 0.170 mmol) and DIPEA (29.6 µL, 0.170 mmol) in dry THF (1.5 mL) was added triphosgene (16.8 mg, 56.7 µmol) in dry DCM (1.5 mL) by dropwise and the mixture was stirred for 1hr at room temperature. To the solution were added **7-5** (44.2 mg, 0.255 mmol) and DIPEA (44.4 µL, 0.255 mmol) in dry DCM (1.5 mL) by dropwise and the mixture was stirred for 2hr at room temperature. The mixture was diluted with chloroform and the organic layer was washed with sat. $NaHCO_3$ aq., water and brine. After removal of the solvent by evaporation, the residue was purified by column chromatography on SiO_2 (chloroform : NH_3 aq. = 300 : 1) to give **7-6** (17.2 mg, 27%) as colorless oil. ^{1}H-NMR (500 MHz, $CDCl_3$) : δ 1.26-1.31 (3H, dd, *J* = 6.5 Hz, 18.5 Hz),1.45 (9H, s), 2.42 (3H, s), 2.39-2.61 (2H, m), 2.90-2.99 (1H, d, *J* = 43.5 Hz), 4.61-4.79 (1H, m), 6.24 (1H, s), 7.09-7.56 (2H, m), 7.56-7.58 (1H, d, *J* = 8.5 Hz). ESI-TOF-MS: m/z for $C_{20}H_{25}NO_6Na$ $[M+Na]^+$: calcd 398.1580, observed 398.1555.

Synthesis of **7**

To a solution of **7-6** (4.3 mg, 11.4 µmol) in dry DCM (2.0 mL) was added TFA (0.4 mL) and the mixture was stirred for 7 hr at 0°C. The solvent was removed by evaporation to give **7** (4.8 mg, quant) as a white solid. **7** was used for evaluating the reaction kinetics of intramolecular cyclization without further purification. ESI-TOF-MS: m/z for $C_{16}H_{17}NO_6Na$ $[M+Na]^+$: calcd 342.0948, observed 342.0953.

Preparation of **8**

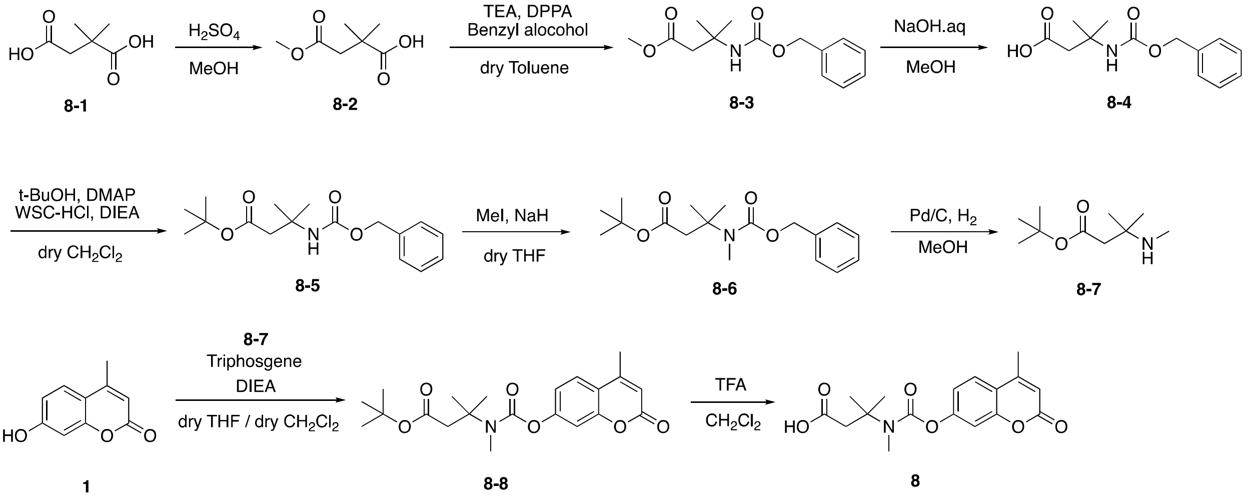

Synthesis of **8-2**

To a solution of **8-1** (2.00 g, 13.7 mmol) in MeOH (20 mL) was added H_2SO_4 (200 μL) and stirred for 18 hr at room temeperature. The solvent was removed by evaporation and the residue was diluted with sat. $NaHCO_3$ aq., followed by wash with hexane. After adjustment of the pH to 1 with 1N HCl aq., the product was extracted with ethyl acetate. The solvent was removed by evaporation to give **8-2** (1.20 g, 55%) as colorless oil. ^{1}H-NMR (500 MHz, $CDCl_3$) : δ 1.30 (6H, s), 2.62 (2H, s), 3.67 (3H, s). ESI-TOF-MS: m/z for $C_7H_{12}O_4Na$ $[M+Na]^+$: calcd 188.0633, observed 183.0654.

Synthesis of **8-3**

To a solution of **8-2** (1.20 g, 7.49 mmol) and triethylamine (1.04 ml, 7.49 mmol) in dry Toluene (10 mL) was added diphenylphosphoryl azide (1.78 ml, 8.24 mmol) and the mixture was refluxed for 25 hr. After removal of the solvent by evaporation, the residue was diluted with ethyl acetate and the organic layer was washed with sat. $NaHCO_3$ aq. and brine. The solvent was removed by evaporation and the mixture was purified by column chromatography on SiO_2 (hexane : ethyl acetate = 10 : 1) to give **8-3** (1.58 g, 80%) as colorless oil. ^{1}H-NMR (500 MHz, $CDCl_3$) : δ 1.41 (6H, s), 2.71 (6H, s), 3.65 (3H, s), 5.06 (2H, s), 5.19 (1H, s), 7.29-7.36 (5H, m). ESI-TOF-MS: m/z for $C_{14}H_{19}O_4Na$ $[M+Na]^+$: calcd 288.1212, observed 288.1245.

Synthesis of **8-4**

To a solution of **8-3** (1.58 g, 5.96 mmol) in MeOH (20 mL) was added 1N NaOH aq. (17.9 mL, 17.9 mmol) and the mixture was stirred for 10 hr at 0°C. After removal of the solvent by evaporation, the residue was diluted with sat. $NaHCO_3$ aq. and the aqueous layer was washed with ether. After adjustment of the pH to 1 with 1N HCl aq., the product was extracted with ethyl acetate. The solvent was removed by evaporation to give **8-4** (1.34 g, 89%) as colorless oil. ^{1}H-NMR (500 MHz, $CDCl_3$) : δ 1.44 (6H, s), 2.78 (2H, s), 5.07 (2H, s), 7.29-7.37 (5H, m). ESI-TOF-MS: m/z for $C_{13}H_{17}O_4Na$ $[M+Na]^+$: calcd 274.1055, observed 274.1091.

Synthesis of **8-5**

To a solution of **8-4** (893 mg, 3.55 mmol), EDC-HCl (1.02 g, 5.33 mmol), DIPEA (1.85 ml, 10.7 mmol) and DMAP (86.9 mg, 0.711 mmol) in dry DCM (15.0 mL) was added *tert*-BuOH (1.69 ml, 17.8 mmol) and the mixture was stirred for 12 hr at room temperature. The mixture was diluted with chloroform and the organic layer was washed with sat. $NaHCO_3$ aq., water and brine. After removal of the solvent by evaporation, the residue was purified by column chromatography on SiO_2 (hexane : ethyl acetate = 10 : 1) to give **8-5** (389 mg, 36%) as colorless oil. ^{1}H-NMR (400 MHz, $CDCl_3$) : δ 1.41 (6H, s), 1.44 (9H, s), 2.55 (2H, s), 5.06 (2H, s), 5.34 (1H, s), 7.30-7.35 (5H, m). ESI-TOF-MS: m/z for $C_{17}H_{25}O_4Na$ $[M+Na]^+$: calcd 330.1681, observed 330.1706.

Synthesis of **8-6**

To a solution of **8-5** (389 mg, 1.27 mmol) and NaH (152 mg, 3.80 mmol) in dry THF (10 mL) was added CH_3I (791 μL, 12.7 mmol) at 0°C and the mixture was stirred for 22 hr at room temperature. The mixture was diluted with ethyl acetate and the organic layer was washed with sat. $NaHCO_3$ aq., water and brine. After removal of the solvent by evaporation, the residue was purified by column chromatography on SiO_2 (hexane : ethyl acetate = 8 : 1) to give **8-6** (224 mg, 55%) as colorless oil. ^{1}H-NMR (500 MHz, $CDCl_3$) : δ 1.41 (9H, s), 1.47 (6H, s), 2.83 (2H, s), 2.97 (3H, s), 5.11 (2H, s), 7.29-7.38 (5H, m). ESI-TOF-MS: m/z for $C_{18}H_{27}NO_4Na$ $[M+Na]^+$: calcd 344.1832, observed 344.1866.

Synthesis of **8-7**

To a solution of **8-6** (224 mg, 0.697 mmol) in MeOH (5.0 mL) was added Pd/C (50.0 mg) and the mixture was stirred for 3.5 hr at room temperature under H_2 atmosphere. After filtration thorough celite, the solvent was removed by evaporation to give **8-7** (93.2 mg, 71%) as white solid and used next reaction without further purification. ^{1}H-NMR (500 MHz, $CDCl_3$) : δ 1.12 (6H, s), 1.44 (9H, s), 2.30 (3H, s), 2.31 (2H, s). ESI-TOF-MS: m/z for $C_{10}H_{21}NO_2Na$ $[M+Na]^+$: calcd 188.1645, observed 188.1662.

Synthesis of **8-8**

To a solution of **1** (31.1 mg, 0.177 mmol) and DIPEA (30.7 μL, 0.177 mmol) in dry THF (1.0 mL) was added triphosgene (17.5 mg, 58.8 μmol) in dry DCM (1.0 mL) by dropwise and the mixture was stirred for 1hr at room temperature. To the solution were added **8-7** (49.6 mg, 0.265 mmol) and DIPEA (46.2 μL, 0.265 mmol) in dry DCM (1.5 mL) by dropwise and the mixture was stirred for 1 hr at room temperature. The mixture was diluted with chloroform and the organic layer was washed with sat. $NaHCO_3$ aq., water and brine. After removal of the solvent by evaporation, the residue was purified by column chromatography on SiO_2 (hexane : ethyl acetate = 2 : 1) to give **8-8** (9.30 mg, 14%) as colorless oil. ^{1}H-NMR (500 MHz, $CDCl_3$) : δ 1.46 (9H, s), 1.53 (6H, s), 2.42 (3H, s), 2.88 (2H, s), 3.12 (3H, s), 6.24 (1H, s), 7.10-7.12 (2H, m), 7.56-7.58 (1H, d, J = 9.0 Hz). ESI-TOF-MS: m/z for $C_{20}H_{24}NO_6Na$ $[M+Na]^+$: calcd 412.1731, observed 412.1696.

Synthesis of **8**

To a solution of **8-8** (3.30 mg, 8.47 μmol) in dry DCM (0.8 mL) was added TFA (0.2 mL) and the mixture was stirred for 7 hr at 0°C. The solvent was removed by evaporation to give **8** (3.5 mg, quant) as a white solid. **8**

was used for evaluating the reaction kinetics of intramolecular cyclization without further purification. Measurement of NMR spectrum of **8** was not conducted due to rapid intramolecular cyclization of **8**. ESI-TOF-MS: m/z for $C_{17}H_{19}NO_6Na$ $[M+Na]^+$: calcd 356.1105, observed 356.1077.

Preparation of **9**

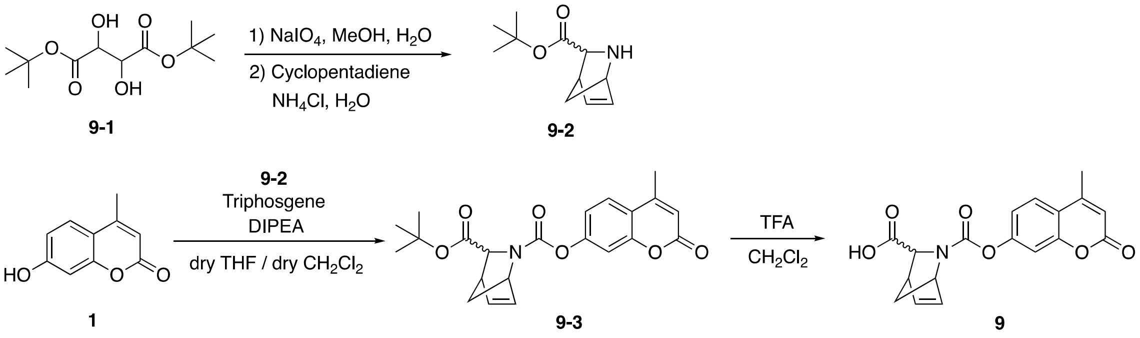

Synthesis of **9-2**

To a solution of **9-1** (100 mg, 0.381 mmol) in MeOH (2.0 mL) was added $NaIO_4$ (97.7 mg, 0.457 mmol) in H2O (1.0 mL) and the mixture was stirred for 70 min at 0 °C. After dilution with H_2O, the product was extracted with ether and the solvent was removed by evaporation. The residue was diluted wit H_2O (0.1 mL) and to the solution were added NH_4Cl (32.2 mg, 0.602 mmol) and cyclopentadiene (127 µL, 1.51 mmol). The mixture was stirred for 18 hr at room temperature, diluted with water, and washed with ether. After adjustment of pH to 12 with 1N NaOH aq., the product was extracted with ethyl acetate to give **9-2** (32.4 mg, 28%) as yellow oil. ^{1}H-NMR (500 MHz, $CDCl_3$) : δ 1.40 (9H, s), 1.58-1.60 (2H, m), 3.37 (1H, s), 3.80 (1H, s), 3.96 (1H, s), 5.84-5.85 (1H, m), 6.26-6.28 (1H, m). ESI-TOF-MS: m/z for $C_{11}H_{18}NO_2Na$ $[M+Na]^+$: calcd 196.1338, observed 196.1351.

Synthesis of **9-3**

To a solution of **1** (20.0 mg, 0.114 mmol) and DIPEA (19.8 µL, 0.114 mmol) in dry THF (1.0 mL) was added triphosgene (11.2 mg, 37.8 µmol) in dry DCM (1.0 mL) by dropwise and the mixture was stirred for 4 hr at room temperature. To the solution were added **9-2** (33.2 mg, 0.170 mmol) and DIPEA (29.7 µL, 0.170 mmol) in dry DCM (1.0 mL) by dropwise and the mixture was stirred for 3 hr at room temperature. The mixture was diluted with chloroform and the organic layer was washed with sat. $NaHCO_3$ aq., water and brine. After removal of the solvent by evaporation, the residue was purified by column chromatography on SiO_2 (hexane : ethyl acetate = 2 : 1) to give **9-3** (17.3 mg, 38%) as colorless oil. ^{1}H-NMR (500 MHz, $CDCl_3$) : δ 1.39-1.41 (9H, d, J=16.0), 1.73-1.77 (2H, m), 2.38 (3H, s), 3.54-3.56 (1H, m), 4.32-4.42 (1H, m), 4.93-5.01 (1H, m), 6.16-6.17 (1H, m), 6.20 (1H, s), 6.56-6.58 (1H, s), 7.08-7.14 (2H, m), 7.51-7.55 (1H, m). ESI-TOF-MS: m/z for $C_{22}H_{23}NO_6Na$ $[M+Na]^+$: calcd 420.1423, observed 420.1388.

Synthesis of **5**

To a solution of **5-3** (4.6 mg, 11.6 µmol) in dry DCM (2.0 mL) was added TFA (0.4 mL) and the mixture was

stirred for 2.5 hr at room temperature. The solvent was removed by evaporation to give **5** (4.0 mg, quant) as a white solid. **9** was used for evaluating the reaction kinetics of intramolecular cyclization without further purification. ^{1}H-NMR (500 MHz, CD_3OD) : δ2.49 (3H, s), 2.54-2.56 (2H, m), 3.35 (1H, s), 5.00-5.02 (1H, d, *J*= 10.0 Hz), 5.44-5.45 (1H, d, *J* = 5.0 Hz), 5.93-5.99 (2H, m), 6.23-6.32 (2H, m), 7.21-7.23 (1H, dd, *J* = 8.8 Hz), 7.24-7.25 (1H, d, *J* = 2.0 Hz), 7.80-7.82 (1H, d, *J* = 8.5 Hz). ESI-TOF-MS: m/z for $C_{18}H_{15}NO_6Na$ $[M+Na]^+$: calcd 364.0797, observed 364.0793.

Preparation of **10**

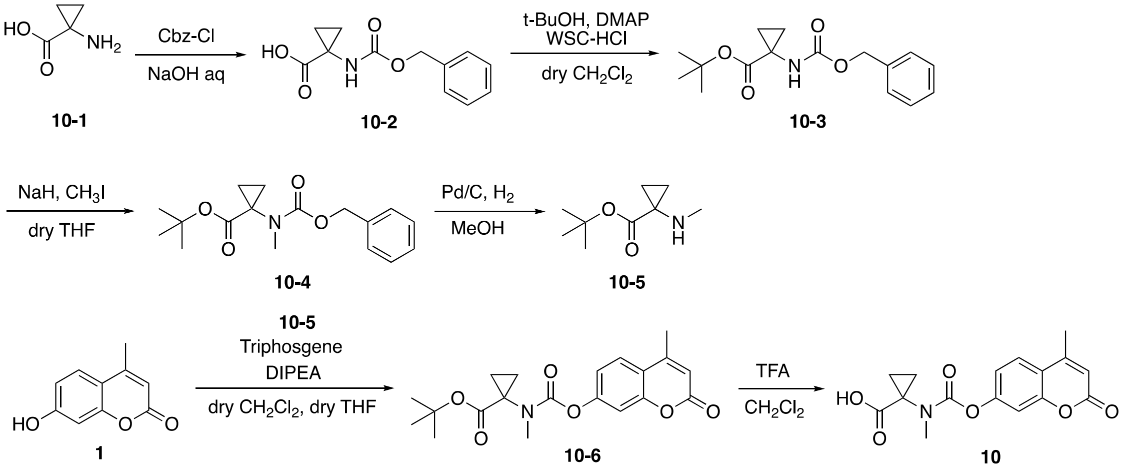

Synthesis of **10-2**

To a solution of **10-1** (745 mg, 7.37 mmol) in 2 N NaOH aq. (10 mL) was added benzyl chloroformate (3.16 mL, 22.1 mmol) and the mixture was stirred for 8 hr at 0°C. The mixture was diluted with water and the aqueous layer was washed with ether. After adjustment of pH to 1 by 5 N HCl aq., the product was extracted with ethyl acetate. The solvent was removed by evaporation to give **10-2** (1.64 g, 95%) as a white solid. ^{1}H-NMR (500 MHz, DMSO-d_6) : δ 0.99-1.32 (4H, m), 5.02 (2H, s), 7.30-7.37 (5H, m), 7.87 (1H, s), 12.41 (1H, s). ESI-TOF-MS: m/z for $C_{12}H_{13}NO_4Na$ $[M+Na]^+$: calcd 258.0742, observed 258.0754.

Synthesis of **10-3**

To a solution of **10-2** (200 mg, 0.85 mmol), EDC-HCl (245 mg, 1.28 mmol), DIPEA (593 μL, 3.41 mmol) and DMAP (20.8 mg, 0.17 mmol) in dry DCM (3.0 mL) was added *tert*-BuOH (122 μL, 1.28 mmol) and the mixture was stirred for 21 hr at room temperature. The mixture was diluted with chloroform and the organic layer was washed with sat. $NaHCO_3$ aq., water and brine. After removal of the solvent by evaporation, the residue was purified by column chromatography on SiO_2 (hexane : ethyl acetate = 3 : 1) to give **10-3** (40.3 mg, 16%) as colorless oil. ^{1}H-NMR (500 MHz, $CDCl_3$) : δ 1.12-1.47 (4H, m) 1.40 (9H, s), 5.11 (1H, s), 7.28-7.37 (5H, m). ESI-TOF-MS: m/z for $C_{16}H_{21}NO_4Na$ $[M+Na]^+$: calcd 314.1368, observed 314.1372.

Synthesis of **10-4**

To a solution of **10-3** (40.3 mg, 0.138 mmol) and NaH (16.6 mg, 0.414 mmol) in dry THF (2.0 mL) was added

CH_3I (86.0 µL, 1.38 mmol) at 0°C and the mixture was stirred for 4.5 hr at room temperature. The mixture was diluted with ethyl acetate and the organic layer was washed with sat. $NaHCO_3$ aq., water and brine. After removal of the solvent by evaporation, the residue was purified by column chromatography on SiO_2 (hexane : ethyl acetate = 3 : 1) to give **10-4** (40.8 mg, 97%) as yellow oil. ^{1}H-NMR (500 MHz, $CDCl_3$) : δ 1.07-1.76 (4H, m), 1.35 (9H, s), 2.94 (3H, s), 5.14 (2H, s), 7.28-7.38 (5H, m). ESI-TOF-MS: m/z for $C_{17}H_{23}NO_4Na$ $[M+Na]^+$: calcd 328.1525, observed 328.1516.

Synthesis of **10-5**

To a solution of **10-4** (81.1 mg, 0.266 mmol) in MeOH (3.0 mL) was added Pd/C (30.0 mg) and the mixture was stirred for 4 hr at room temperature under H_2 atmosphere. After filtration thorough celite, the solvent was removed by evaporation to give **10-5** (56.6 mg, quant) as white solid and used next reaction without further purification. ^{1}H-NMR (500 MHz, $CDCl_3$) : δ 0.87-1.16 (4H, m) 1.43 (9H, s), 2.06 (1H, s), 2.41 (3H, s). ESI-TOF-MS: m/z for $C_9H_{18}NO_2Na$ $[M+Na]^+$: calcd 172.1338, observed 172.1303.

Synthesis of **10-6**

To a solution of **1** (34.6 mg, 0.197 mmol) and DIPEA (34.3 µL, 0.197 mmol) in dry THF (1.5 mL) was added triphosgene (19.4 mg, 65.5 µmol) in dry DCM (1.5 mL) by dropwise and the mixture was stirred for 2 hr at room temperature. To the solution were added **10-5** (40.4 mg, 0.236 mmol) and DIPEA (41.1 µL, 0.236 mmol) in dry DCM (1.5 mL) by dropwise and the mixture was stirred for 1 hr at room temperature. The mixture was diluted with chloroform and the organic layer was washed with sat. $NaHCO_3$ aq., water and brine. After removal of the solvent by evaporation, the residue was purified by column chromatography on SiO_2 (chloroform) to give **10-6** (14.9 mg, 20%) as colorless oil. ^{1}H-NMR (500 MHz, $CDCl_3$) : δ 1.20-2.04 (4H, m) 1.47 (9H, s), 2.43 (3H, s), 3.02 (3H, s), 6.24 (1H, s), 7.08-7.14 (1H, m), 7.11 (1H, s), 7.57-7.59 (1H, d, J = 8.2 Hz). ESI-TOF-MS: m/z for $C_{20}H_{23}NO_6Na$ $[M+Na]^+$: calcd 396.1423, observed 396.1389.

Synthesis of **10**

To a solution of **10-6** (5.0 mg, 13.4 µmol) in dry DCM (1.5 mL) was added TFA (0.3 mL) and the mixture was stirred for 7 hr at room temperature. The solvent was removed by evaporation to give **10** (5.2 mg, quant) as a white solid. **10** was used for evaluating the reaction kinetics of intramolecular cyclization without further purification. ESI-TOF-MS: m/z for $C_{16}H_{15}NO_6Na$ $[M+Na]^+$: calcd 340.0792, observed 340.0790.

Preparation of **11**

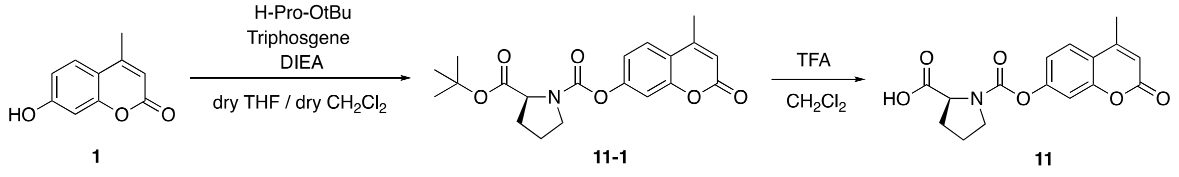

Synthesis of **11-1**

To a solution of **1** (50.0 mg, 0.284 mmol) and DIPEA (49.5 µL, 0.284 mmol) in dry THF (2.0 mL) was added triphosgene (28.1 mg, 94.7 µmol) in dry DCM (2.5 mL) by dropwise and the mixture was stirred for 3 hr at room temperature. To the solution were added L-Proline *tert*-butyl ester (72.9 mg, 0.426 mmol) and DIPEA (74.2 µL, 0.426 mmol) in dry DCM (2.5 mL) by dropwise and the mixture was stirred for 1.5 hr at room temperature. The mixture was diluted with chloroform and the organic layer was washed with sat. $NaHCO_3$ aq., water and brine. After removal of the solvent by evaporation, the residue was purified by column chromatography on SiO_2 (hexane : ethyl acetate = 2 : 1) to give **11-1** (45.6 mg, 43%) as colorless oil. ^{1}H-NMR (500 MHz, $CDCl_3$) : δ 1.45-1.47 (9H, d, *J* = 9.0 Hz), 1.92-2.35 (4H, m), 2.41 (3H, d, *J* = 1.0 Hz), 3.48-3.79 (2H, m), 4.31-4.41 (1H, m), 6.23 (1H, d, *J* = 1.0 Hz), 7.09-7.16 (2H, m), 7.55-7.57 (1H, d, *J* = 9.0 Hz). ESI-TOF-MS: m/z for $C_{20}H_{23}NO_6Na$ $[M+Na]^+$: calcd 396.1423, observed 396.1388.

Synthesis of **11**

To a solution of **11-1** (22.8 mg, 61.1 µmol) in dry DCM (2.0 mL) was added TFA (0.4 mL) and the mixture was stirred for 7 hr at room temperature. After removal of the solvent by evaporation, the residue was purified by column chromatography on SiO_2 (Chloroform : MeOH : NH_3 aq. = 100 : 10 : 1) to give **11** (20.3 mg, quant) as colorless oil. **11** was used for evaluating the reaction kinetics of intramolecular cyclization without further purification. ^{1}H-NMR (500 MHz, MeOD) : δ 2.00-2.46 (4H, m), 2.47 (3H, s), 3.53-3.78 (2H, m), 4.39-4.58 (1H, m), 6.29 (1H, s), 7.12-7.22 (2H, m), 7.76-7.79 (1H, m). ESI-TOF-MS: m/z for $C_{16}H_{15}NO_6Na$ $[M+Na]^+$: calcd 340.0797, observed 340.0786.

Preparation of **12**

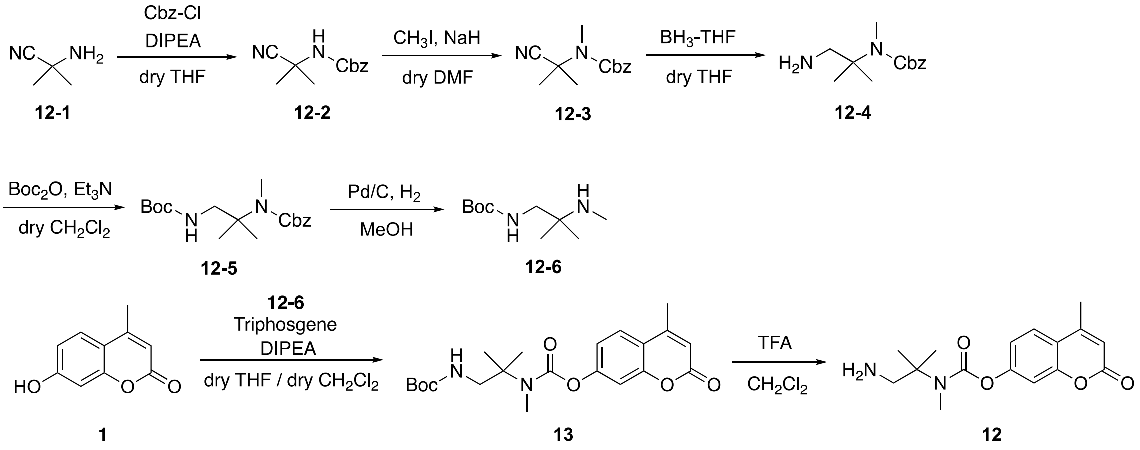

Synthesis of **12-2**

To a solution of **12-1** (800 mg, 9.51 mmol) and DIPEA (2.13 mL, 12.4 mmol) in dry THF (20 mL) was added benzyl chloroformate (1.61 mL, 11.4 mmol) and the mixture was stirred for 2.5 hr at 0 °C. After removal of the solvent, the residue was diluted with ethyl acetate and the organic layer was washed with water and brine. After removal of the solvent, the crude product was purified by column chromatography on SiO_2 (Hexane : Chloroform = 10 : 1 → 0 : 1) to give **12-2** (1.81 g, 87%) as colorless oil. ^{1}H-NMR(500 MHz, $CDCl_3$): δ 1.69 (6H, s), 4.98 (1H, s), 5.14 (2H, s), 5.06 (2H, s), 7.37-7.32 (5H, m), ESI-TOF-MS: m/z for $C_{12}H_{14}N_2O_2Na[M+Na]^+$: calcd 241.0953, observed 241.0951.

Synthesis of **12-3**

To a solution of **12-2** (1.80 g, 8.29 mmol) in dry DMF (10 mL) was added and NaH (199 mg, 8.29 mmol) and the mixture was stirred for 10 min at 0°C. To the solution was added CH_3I (4.13 mL, 66.3 mmol) and the mixture was stirred for 16 hr at 0°C. After quenching the reaction by addition of water, the mixture was extracted with ethyl acetate and the organic layer was washed with water and brine. After removal of the solvent by evaporation, the residue was purified by column chromatography on SiO_2 (hexane : ethyl acetate = 5 : 1) to give **12-3** (1.35 g, 70%) as brown oil. ^{1}H-NMR (500 MHz, $CDCl_3$): δ1.74 (6H, s), 2.99 (3H, s), 5.19 (2H, s), 7.41-7.26 (5H, m). ESI-TOF-MS: m/z for $C_{13}H_{16}N_2O_2Na$ $[M+Na]^+$: calcd 255.1109, observed 255.1130.

Synthesis of **12-4**

To a solution of **12-3** (750 mg, 3.22 mmol) in dry THF (20 mL) was added BH_3-THF (20.0 mL, 6.37 mmol) and the mixture was stirred for 17 hr at room temperature. After quenching the reaction by addition of MeOH, 1N HCl (15 mL) was added and the mixture was refluxed for 1hr. After removal of THF and methanol by evaporation, the aqueous layer was washed with chloroform. After adjustment of the pH to 12 with 1N NaOH aq., the crude product was extracted with chloroform and purified by column chromatography on SiO_2 (chloroform : methanol : NH_3 aq. = 100 : 10 : 1) to give **12-4** (140 mg, 18 %) as brown oil. ^{1}H-NMR (500 MHz, $CDCl_3$): δ1.09 (6H, s), 2.31 (3H, s), 3.14 (2H, d, J = 5.5 Hz), 5.10 (2H, s), 7.36-7.26 (5H, m). ESI-TOF-MS: m/z for $C_{13}H_{21}N_2O_2$ $[M+H]^+$: calcd 237.1603, observed 237.1589.

Synthesis of **12-5**

To a solution of **12-4** (40 mg, 0.169 mmol) and Et_3N (46.9 µL, 0.339 mmol) in dry DCM (3 mL) was added Boc_2O (40.6 mg, 0.186 mmol) and the mixture was stirred for 23 hr at room temperature. After dilution with DCM, the mixture was washed with water and brine. After removal of the solvent, the crude product was purified by column chromatography on SiO_2 (hexane : ethyl acetate = 5 : 1 to 1 : 1) to give **12-5** (43.1 mg, 76 %) as colorless oil. ^{1}H-NMR(500 MHz, $CDCl_3$): δ1.20 (6H, s), 1.44 (9H, s), 2.83 (3H, s), 3.58 (2H, d, J = 6.5 Hz), 5.10 (2H, s), 5.19 (1H, s), 7.36-7.30 (5H, m). ESI-TOF-MS: m/z for $C_{18}H_{28}N_2O_4Na$ $[M+Na]^+$: calcd 359.1947, observed 359.1857.

Synthesis of **12-6**

To a solution of **12-5** (40 mg, 119 µmol) in MeOH (2.0 mL) was added Pd/C (8 mg) and the mixture was stirred for 5.5 hr at room temperature under H_2 atmosphere. After removal of Pd/C by filtration through celite, the solvent was removed by evaporation to give **12-6** (19.4 mg, 81 %) as a colorless oil. ^{1}H-NMR (500 MHz, $CDCl_3$): δ1.30 (6H, s), 1.46 (9H, s), 2.89 (3H, s), 2.95 (2H, s). ESI-TOF-MS: m/z for $C_{10}H_{23}N_2O_2$ $[M+H]^+$: calcd 203.1760, observed 203.1779.

Synthesis of **13**

To a solution of **1** (13.2 mg, 75.0 µmol) and DIPEA (12.8 µL, 74 µmol) in dry THF (1.0 mL) was added triphosgene (6.6 mg, 22.2 µmol) in dry DCM (1.0 mL) by dropwise and the mixture was stirred for 6 hr at room temperature. To the solution were added **12-6** (18.2 mg, 90.0 µmol) and DIPEA (19.2 µL, 111 µmol) in dry DCM (2.5 mL) by dropwise and the mixture was stirred for 2 hr at room temperature. The mixture was diluted with chloroform and the organic layer was washed with sat. $NaHCO_3$ aq., water and brine. After removal of the solvent by evaporation, the residue was purified by column chromatography on SiO_2 (chloroform : methanol = 100 : 1 → 50 : 1) to give **12-7** (11.8 mg, 39 %) as colorless oil. ^{1}H-NMR (500 MHz, $CDCl_3$): δ1.36 (6H, s), 1.48 (9H, s), 2.41 (3H, s), 2.87 (3H, s), 3.65-3.63 (2H, d, J = 6.5 Hz), 5.87 (1H, s), 6.23 (1H, s), 7.13-7.11 (1H, dd, J = 2.0 Hz, 8.5 Hz), 7.15-7.14 (1H, d, J = 2.0 Hz), 7.57-7.55 (1H, d, J = 8.5 Hz). ESI-TOF-MS: m/z for $C_{21}H_{28}N_2O_6Na$ $[M+Na]^+$: calcd 427.1845, observed 427.1861.

Synthesis of **12**

To a solution of **13** (5.0 mg, 12.4 µmol) in dry DCM (1.5 mL) was added TFA (0.3 mL) and the mixture was stirred for 7 hr at room temperature. The solvent was removed by evaporation to give **12** (5.0 mg, quant) as a white solid. **12** was used for evaluating the reaction kinetics of intramolecular cyclization without further purification. Measurement of NMR spectrum of **12** was not conducted due to rapid intramolecular cyclization of **12**. ESI-TOF-MS: m/z for $C_{16}H_{21}N_2O_4$ $[M+H]^+$: calcd 305.1501, observed 305.1491.

Preparation of **14**

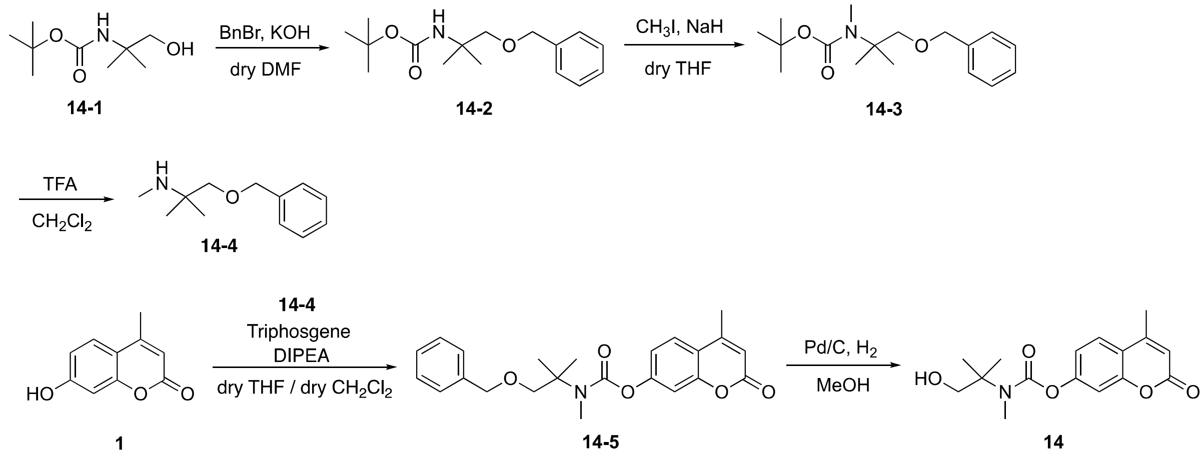

Synthesis of **14-2**

To a solution of **14-1** (635 mg, 3.36 mmol) and KOH (1.3 g, 16.8 mmol) in dry DMF (10 mL) was added benzyl bromide (1.2 mL, 10.1 mmol) and the mixture was stirred for 3hr at room temperature. After removal of the solvent by evaporation, the mixture was diluted with ethyl acetate and the organic layer was washed with water and brine. The solvent was removed by evaporation and the residue was purified by column chromatography on SiO_2 (hexane : ethyl acetate = 30 : 1) to give **14-2** (600 mg, 64%) as colorless oil. ^{1}H-NMR (500 MHz, $CDCl_3$) : δ 1.30 (6H, s), 1.41 (9H, s), 3.40 (2H, s), 4.54 (2H, s), 7.29-7.35 (5H, m). ESI-TOF-MS m/z for $C_{16}H_{25}NO_3Na$ $[M+Na]^+$: calcd 302.1732, observed 302.1744.

Synthesis of **14-3**

To a solution of **14-2** (600 mg, 2.15 mmol) and NaH (260 mg, 6.45 mmol) in dry THF (5.0 mL) was added CH_3I (1.3 mL, 21.5 mmol) and the mixture was stirred for 13 hr at room temperature. After quenching the reaction by addition of water, the mixture was diluted with ethyl acetate and the organic layer was washed with water and brine. After removal of the solvent by evaporation, the residue was purified by column chromatography on SiO_2 (hexane : ethyl acetate = 20 : 1) to give **14-3** (506 mg, 80%) as colorless oil. ^{1}H-NMR (400 MHz, $CDCl_3$) : δ 1.38 (6H, s), 1.44 (9H, s), 2.93 (3H, s), 3.64 (2H, s), 4.52 (2H, s), 7.25-7.36 (5H, m). ESI-TOF-MS m/z for $C_{17}H_{27}NO_3Na$ $[M+Na]^+$: calcd 316.1889, observed 316.1906.

Synthesis of **14-4**

To a solution of **14-3** (506 mg, 1.72 mmol) in dry DCM (5.0 mL) was added TFA (2.5 mL) and the mixture was stirred for 1 hr at room temperature. After removal of the solvent by evaporation, the residue was diluted with ethyl acetate. The organic layer was washed with sat. $NaHCO_3$ aq. and brine to give **14-4** (277 mg, 83%) as yellow oil. ^{1}H-NMR (400 MHz, $CDCl_3$) : δ 1.35 (6H, s), 2.56-2.60 (3H, t, J = 5.6 Hz), 3.41 (2H, s), 4.57 (2H, s), 7.26-7.39 (5H, m), 9.67 (1H, s). ESI-TOF-MS: m/z for $C_{12}H_{19}NO$ $[M+H]^+$: calcd 194.1545, observed 194.1561.

Synthesis of **14-5**

To a solution of **1** (65.0 mg, 0.37 mmol) and DIPEA (65.0 μL, 0.37 mmol) in dry THF (2.0 mL) was added triphosgene (41.0 mg, 0.12 mmol) in dry DCM (2.0 mL) by dropwise and the mixture was stirred for 1.5 hr at room temperature. To the solution were added **14-4** (96.4 mg, 0.56 mmol) and DIPEA (97.5 μL, 0.56 mmol) in dry DCM (2.0 mL) by dropwise and the mixture was stirred for 1 hr at room temperature. The mixture was diluted with chloroform and the organic layer was washed with sat. $NaHCO_3$ aq., water and brine. After removal of the solvent by evaporation, the residue was purified by column chromatography on SiO_2 (hexane : ethyl acetate = 2 : 1) to give **14-5** (48.6 mg, 33%) as colorless oil. ^{1}H-NMR (400 MHz, $CDCl_3$) : δ 147 (6H, s), 2.42 (3H, s), 3.15 (3H, s), 3.70 (2H, s), 4.56 (2H, s), 6.24 (1H, s), 7.03-7.07 (2H, m), 7.27-7.38 (5H, m), 7.54-7.57 (1H, d, J = 8.8 Hz). ESI-TOF-MS: m/z for $C_{23}H_{25}NO_5Na$ $[M+Na]^+$: calcd 418.1630, observed 418.1612.

Synthesis of **14**

To a solution of **14-6** (4.3 mg, 0.011 mmol) in MeOH (1.0 mL) was added Pd/C (5.0 mg) under N_2 atmosphere and the mixture was stirred for 9 hr at 0 °C under H_2 atmosphere. Pd/C was removed by filtration through celite to give **14** (3.3 mg, quant). **14** was used for evaluating the reaction kinetics of intramolecular cyclization without further purification. ESI-TOF-MS: m/z for $C_{16}H_{19}NO_5Na$ $[M+Na]^+$: calcd 328.1161, observed 328.1142.

Preparation of **15** and **16**

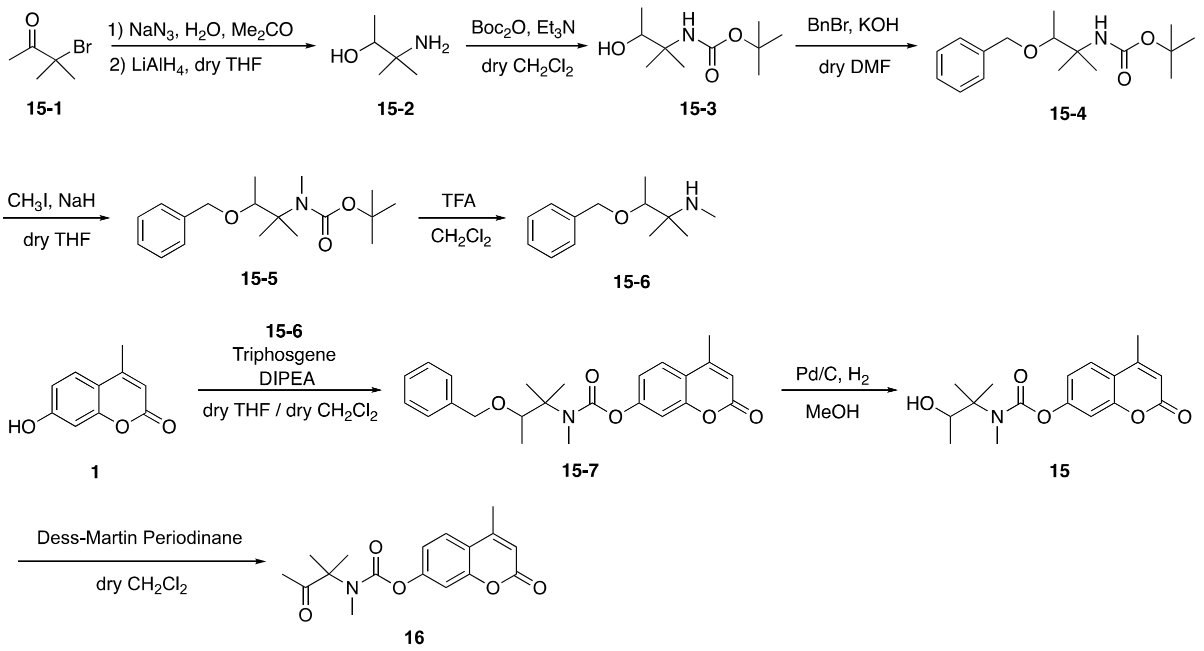

Synthesis of **15-2**

To a solution of **15-2** (1.0 g, 6.06 mmol) in acetone (50 mL) and water (5 mL) was added NaN_3 (394.0 mg, 6.06 mmol) and the mixture was refluxed for 17 hr. After removal of the solvent by evaporation, the residue

was diluted with ethyl acetate and the organic layer was washed with water and brine. The solvent was removed by evaporation at 20 °C and the mixture was diluted with dry THF (30 mL). To the solution was added $LiAlH_4$ (414.0 mg, 10.91 mmol) and the mixture was stirred for 6.5 hr at room temperature. The reaction was quenched by addition of water and the precipitate was filtrated thorough celite. After removal of THF by evaporation, the product was extracted with chloroform to give **15-2** (422.0 mg, 67%) as yellow oil.^{1}H-NMR (400 MHz, $CDCl_3$) : δ 1.02 (3H, s), 1.11 (6H, s), 3.39-3.45 (1H, m), 1.02 (3H, s). ESI-TOF-MS: m/z for $C_5H_{13}NONa$ $[M+Na]^+$: calcd 126.0895, observed 126.0897.

Synthesis of **15-3**

To a solution of **15-2** (422.0 mg, 4.1 mmol) and triethyl amine (630.0 µL, 4.5 mmol) in dry CH_2Cl_2 (5 mL) was added Boc_2O (984.4 mg, 4.5 mmol) and the mixture was stirred for 23 hr at room temperature. After removal of the solvent, the mixture was diluted with 1N citric acid aq. and extracted with ethyl acetate. The crude product was purified by column chromatography on SiO_2 (hexane : ethyl acetate = 3 : 1) to give **15-3** (292.5 mg, 35%) as colorless oil. ^{1}H-NMR (400 MHz, $CDCl_3$) : δ 1.14-1.18 (6H, m), 1.34 (3H, s), 1.44 (9H, s), 3.69-3.74 (1H, q, J = 6.4 Hz), 4.63 (1H, s). ESI-TOF-MS: m/z for $C_1H_{21}NO_3Na$ $[M+Na]^+$: calcd 226.1419, observed 226.1446.

Synthesis of **15-4**

To a solution of **15-3** (292.5 mg, 1.44 mmol) and KOH (557.1 mg, 7.20 mmol) in dry DMF (4 mL) was added benzyl bromide (513.3 µL, 4.32 mmol) and the mixture was stirred for 3hr at room temperature. After removal of the solvent by evaporation, the mixture was diluted with ethyl acetate and the organic layer was washed with water and brine. The solvent was removed by evaporation and the residue was purified by column chromatography on SiO_2 (hexane : ethyl acetate = 3 : 1) to give **15-4** (344.0 mg, 81%) as colorless oil. ^{1}H-NMR (500 MHz, $CDCl_3$) : δ 1.15-1.16 (3H, d, J = 6.5 Hz), 1.29 (6H, s), 1.42 (9H, s), 3.71-3.72 (1H, d, J = 5.5 Hz), 4.43-4.64 (2H, m), 4.78 (1H, s), 7.27-7.32 (5H, m). ESI-TOF-MS: m/z for $C_{17}H_{27}NO_3Na$ $[M+Na]^+$: calcd 316.1889, observed 316.1916.

Synthesis of **15-5**

To a solution of **15-4** (344.0 mg, 1.17 mmol) and NaH (141.1 mg, 3.51 mmol) in dry THF (4 mL) was added CH_3I (707.4 µL, 11.7 mmol) and the mixture was stirred for 19 hr at 40°C. After quenching the reaction by addition of water, the mixture was diluted with ethyl acetate and the organic layer was washed with water and brine. After removal of the solvent by evaporation, the residue was purified by column chromatography on SiO_2 (hexane : ethyl acetate = 20 : 1) to give **15-5** (217.3 mg, 60%) as colorless oil. ^{1}H-NMR (500 MHz, $CDCl_3$) : δ 1.08-1.10 (3H, d, J = 6.5 Hz), 1.34-1.39 (6H, d, J = 23 Hz), 1.45 (9H, s), 2.92 (3H, s), 4.41-4.58 (3H, m), 7.24-7.33 (5H, m). $C_{18}H_{29}NO_3Na$ $[M+Na]^+$: calcd 330.2045, observed 330.2049.

Synthesis of **15-6**

To a solution of **15-5** (217.3 mg, 0.71 mmol) in dry DCM (4 mL) was added TFA (1 mL) and the mixture was stirred for 2.5 hr at room temperature. After removal of the solvent by evaporation, the residue was diluted with ethyl acetate. The organic layer was washed with sat. $NaHCO_3$ aq. and brine to give **15-6** (150.1 mg,

quant) as yellow oil. ^{1}H-NMR (500 MHz, $CDCl_3$) : δ 1.03-1.06 (6H, d, *J* = 11.5 Hz), 1.17-1.18 (3H, d, *J* = 6.0 Hz), 2.28 (3H, s), 3.42-3.46 (1H, q, *J* = 6.5 Hz), 4.39-4.67 (2H, m), 7.27-7.52 (5H, m). ESI-TOF-MS: m/z for $C_{13}H_{22}NO$ $[M+H]^+$: calcd 208.1701, observed 208.1699.

Synthesis of **15-7**

To a solution of **1** (35.1 mg, 0.20 mmol) and DIPEA (35.1 µL, 0.20 mmol) in dry THF (1.0 mL) was added triphosgene (22.3 mg, 0.067 mmol) in dry DCM (1.0 mL) by dropwise and the mixture was stirred for 2.5 hr at room temperature. To the solution were added **15-6** (62.1 mg, 0.30 mmol) and DIPEA (52.7 µL, 0.30 mmol) in dry DCM (1.5 mL) by dropwise and the mixture was stirred for 1 hr at room temperature. The mixture was diluted with chloroform and the organic layer was washed with sat. $NaHCO_3$ aq., water and brine. After removal of the solvent by evaporation, the residue was purified by column chromatography on SiO_2 (hexane : ethyl acetate = 2 : 1) to give **15-7** (26.4 mg, 32%) as colorless oil. ^{1}H-NMR (500 MHz, $CDCl_3$) : δ 1.16-1.17 (3H, d, *J* = 6.5 Hz), 1.45-1.47 (6H, d, *J* = 12.5 Hz), 2.42 (3H, s), 3.14 (1H, s), 4.44-4.64 (3H, m), 6.24 (1H, s), 7.00-7.04 (2H, m), 7.26-7.34 (5H, m), 7.55-7.56 (1H, d, *J* = 8.5 Hz). ESI-TOF-MS: m/z for $C_{24}H_{27}NO_5Na$: calcd 432.1787 $[M+Na]^+$, observed 432.1786.

Synthesis of **15**

To a solution of **15-7** (5.0 mg, 12.0 µmol) in MeOH (1.0 mL) was added Pd/C (4.0 mg) under N_2 atmosphere and the mixture was stirred for 2 hr at 0 °C under H_2 atmosphere. Pd/C was removed by filtration through celite to give **15** (3.8 mg, quant). **15** was used for evaluating the reaction kinetics of intramolecular cyclization without further purification. ESI-TOF-MS: m/z for $C_{17}H_{21}NO_5Na$: calcd 342.1317 $[M+Na]^+$, observed 342.1327.

Synthesis of **16**

To a solution of **15-7** (11.2 mg, 0.035 mmol, 1.0 eq) in dry MeOH (1.0 mL) was added Pd/C (5.0 mg) and the mixture was stirred for 31 hr at room temperature under H_2 atomosphere. After removal of Pd/C through celite, the solvent was removed by evaporation without heating. The residue was diluted with dry CH_2Cl_2 (1.0 mL) and to the solution was added Dess-Martin Periodinane (23.8 mg, 0.056 mmol). The mixture was stirred for 2hr at room temperature. After dilution with CH_2Cl_2, the organic layer was washed with sat. $NaHCO_3$ aq. and brine. The crude product was purified by column chromatography on SiO_2 (hexane : ethyl acetate = 1 : 2), followed by further purification with HPLC (YMC-Triart C18, 250×10 mmI.D., Flow rate; 3.0 mL/min, detection UV (220 nm), mobile phase gradient : CH_3CN (0.1% TFA) / H_2O (0.1% TFA) = 30/70 → 70/30, linear gradient over 40 min) to give **16** (3.4 mg, 31%) as a white solid. ^{1}H-NMR (500 MHz, $CDCl_3$) : δ 1.44 (6H, s), 2.18 (3H, s), 2.41 (3H, s), 3.16 (3H, s), 6.24 (1H, s), 7.08-7.11 (2H, m), 7.56-7.57 (1H, d, *J* = 8.5 Hz). ESI-TOF-MS: m/z for $C_{17}H_{20}NO_5$: calcd 318.1336 $[M+H]^+$, observed 318.1375.

Preparation of **18**

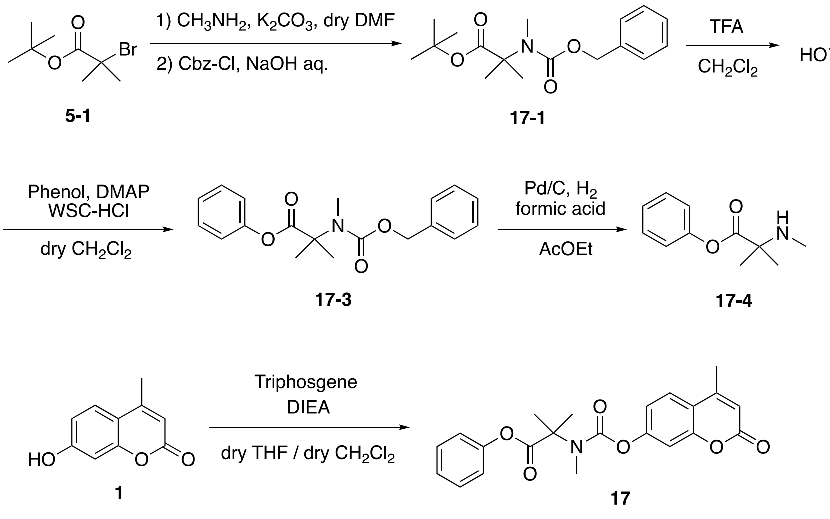

Synthesis of **17-1**

To a solution of **5-1** (1.00 g, 4.50 mmol) and K_2CO_3 (1.87 g, 13.5 mmol) in dry DMF (15.0 mL) was added methylamine (665 μL, 6.75 mmol) and the mixture was stirred for 45 hr at 60 °C. After dilution with 1N citric acid aq., the aqueous layer was washed with chloroform. The pH of the aqueous layer was adjusted to pH 12 with 5 N NaOH aq. and the product was extracted with ether. The solvent was removed by evaporation without heating and the residue was diluted with 2N NaOH aq. (15 mL). To the solvent was added benzyl chloroformate (2.27 mL, 13.5 mmol) and the mixture was stirred for 1.5 hr at 0 °C. After dilution with ethyl acetate, the organic layer was washed with sat. $NaHCO_3$ aq., water and brine. After removal of the solvent by evaporation, the crude product was purified by column chromatography on SiO_2 (hexane : ethyl acetate = 10 : 1) to give **17-1** (856 mg, 62%) as colorless oil. ^{1}H-NMR (400 MHz, $CDCl_3$) : δ 1.34 (9H, s), 1.42 (6H, s), 2.96 (3H, s), 5.12 (2H, s), 7.25-7.34 (5H, m). ESI-TOF-MS: m/z for $C_{19}H_{21}NO_4Na$: calcd 330.1681 $[M+Na]^+$, observed 330.1684.

Synthesis of **17-2**

To a solution of **17-1** (238.8 mg, 0.78 mmol) in dry DCM (5 mL) was added TFA (5 mL) and the mixture was stirred for 1.5 hr at room temperature. After removal of the solvent by evaporation, the residue was diluted with 1N NaOH aq. and the aqueous layer was washed with ethyl acetate. After adjustment of pH of the aqueous layer to 2 with 1N HCl, the product was extracted with ethyl acetate, followed by removal of the solvent by evaporation to give **17-2** (161.4 mg, 82%) as a white solid. ^{1}H-NMR (400 MHz, $CDCl_3$) : δ 1.42 (6H, s), 2.91 (3H, s), 5.05 (2H, s), 7.23-7.29 (2H, m), 7.99 (3H, s). ESI-TOF-MS: m/z for $C_{13}H_{17}NO_4Na$: calcd 274.1050 $[M+Na]^+$, observed 274.0990.

Synthesis of **17-3**

To a solution of **17-2** (100 mg, 0.398 mmol), EDC-HCl (14.6 mg, 0.597 mmol) and DMAP (10.5 mg, 0.0796 mmol) in dry DCM (3.0 mL) was added phenol (52.5 μl, 0.597 mmol) and the mixture was stirred for 7 hr at room temperature. The mixture was diluted with ethyl acetate and the organic layer was washed with sat.

$NaHCO_3$ aq., water and brine. After removal of the solvent by evaporation, the residue was purified by column chromatography on SiO_2 (hexane : ethyl acetate = 3 : 1) to give **17-3** (66.9 mg, 51%) as colorless oil. ^{1}H-NMR (500 MHz, $CDCl_3$) : δ 1.62 (6H, s), 3.06 (3H, s), 5.21 (2H, s), 7.06-7.40 (10H, m). ESI-TOF-MS: m/z for $C_{19}H_{21}NO_4Na$: calcd 350.1368 $[M+Na]^+$, observed 1376.

Synthesis of **17-4**

To a solution of **17-3** (46.3 mg, 0.141 mmol) and formic acid (5 drops) in MeOH (2.0 mL) was added Pd/C (20.0 mg) under N_2 atmosphere and the mixture was stirred for 1 hr at 0 °C under H_2 atmosphere. After removal of Pd/C by filtration through celite, the solvent was removed by evaporation without heating to give **17-4** (27.2 mg, quant). **17-4** was used to the next reaction without further purification due to the instability of **17-4**,. ESI-TOF-MS: m/z for $C_{11}H_{16}NO_2$: calcd 194.1181 $[M+H]^+$, observed 194.1187.

Synthesis of **17**

To a solution of **1** (24.9 mg, 0.141 mmol) and DIPEA (36.9 µl, 0.212 mmol) in dry THF (1.0 mL) was added triphosgene (14.0 mg, 47.1 µmol) in dry DCM (1.0 mL) by dropwise and the mixture was stirred for 5.5 hr at room temperature. To the solution were added **17-4** (62.1 mg, 0.30 mmol) and DIPEA (52.7 µL, 0.30 mmol) in dry DCM (1.5 mL) by dropwise and the mixture was stirred for 1 hr at room temperature. The mixture was diluted with chloroform and the organic layer was washed with sat. $NaHCO_3$ aq., water and brine. After removal of the solvent by evaporation, the residue was purified by column chromatography on SiO_2 (hexane : ethyl acetate = 1 : 1) to give **17** (4.2 mg, 8%) as colorless oil. ^{1}H-NMR (500 MHz, $CDCl_3$) : δ 1.68 (6H, s), 2.42 (3H, s), 3.22 (3H, s), 6.25 (1H, s), 6.81-7.58 (8H, m). ESI-TOF-MS: m/z for $C_{22}H_{22}NO_6$: calcd 396.1447 $[M+H]^+$, observed 396.1453.

Preparation of **18**

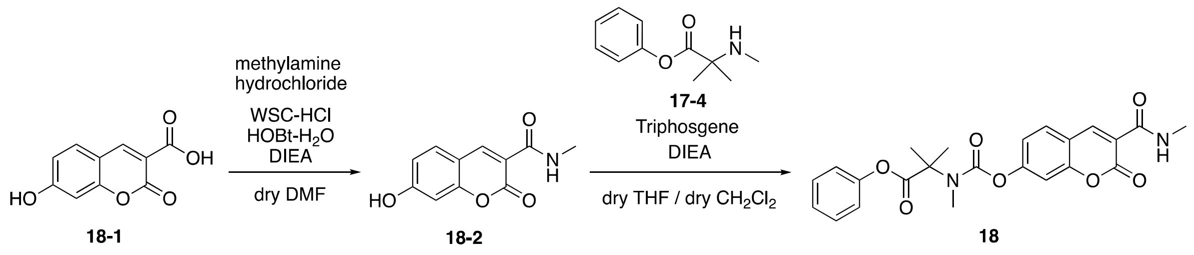

Synthesis of **18-2**

To a solution of **18-1**[S2] (200 mg, 0.970 mmol), HOBt-H_2O (223 mg, 1.46 mmol), WSC-HCl (279 mg, 1.46 mmol) and DIPEA (843 µL, 4.85 mmol) in dry DMF (5 mL) was added methylamine hydrochloride (98.2 mg, 1.46 mmol) and the mixture was stirred overnight at room temperature. After removal of the solvent, the residue was washed with acetone and further purified by column chromatography on SiO_2 (chloroform : methanol = 40 : 1) to give **18-2** (191 mg, 90%) as a white solid. ^{1}H-NMR (500 MHz, DMSO-d_6) : δ 2.83 (3H, J = 4.5 Hz, d), 6.78 (1H, s), 6.86 (1H, J = 10.5 Hz, d), 7.80 (1H, J = 9.0 Hz, d), 8.85-8.57 (1H, brs), 8.76 (1H, s). ESI-TOF-MS: m/z for $C_{11}H_{10}NO_4$: calcd 220.0610 $[M+H]^+$, observed 220.0621.

Synthesis of **18**

To a solution of **18-2** (22.6 mg, 0.103 mmol) and DIPEA (26.9 µl, 0.155 mmol) in dry DCM (0.5 mL) and dry THF (0.5 mL) was added triphosgene (10.0 mg, 34.0 µmol) the mixture was stirred for 1 hr at 0 °C. To the solution were added **17-4** (20.0 mg, 0.103 mmol) and the mixture was stirred for 0.5 hr at 0 °C. The mixture was diluted with ethyl acetate and the organic layer was washed with water and brine. After removal of the solvent by evaporation, the residue was purified by column chromatography on SiO_2 (hexane : ethyl acetate = 1 : 1) to give **18** (11.2 mg, 25%) as a white solid. ^{1}H-NMR (500 MHz, $CDCl_3$) : δ 1.69 (6H, brs), 3.02 (3H, *J* = 5.0 Hz, d), 3.22 (3H, brs), 7.06-7.12 (1H, m), 7.16-7.19 (1H, m), 7.19-7.24 (2H, m). 7.34-7.38 (3H, m), 7.66 (1H, *J* = 8.0 Hz, d), 8.66-8.72 (1H, m), 8.88 (1H, s). ESI-TOF-MS: m/z for $C_{23}H_{23}N_2O_7Na$: calcd 461.1325 $[M+Na]^+$, observed 461.1323.

References

S1. Sepulceda, B.; Quispe, C.; Simirgiotis, M.; Torres-Benítez, A.; Reyes-Ortíz, J.; Areche, C.; García-Beltrán, O.; *Bioorg. Med. Chem. Lett.* **2016**, *26*, 5732-5735.

S2. Guo, P.; Chen, Q.; Liu, T.; Yang, Q.; Qian, X.; *ACS Med. Chem. Lett.* **2013**, *4*, 527-531.
